# Efficacy and safety of stem cell therapy in cerebral palsy: A systematic review and meta-analysis

**DOI:** 10.3389/fbioe.2022.1006845

**Published:** 2022-12-14

**Authors:** Jiayang Qu, Lin Zhou, Hao Zhang, Dongmiao Han, Yaolin Luo, Junming Chen, Lincai Li, Zhengwei Zou, Zhengyi He, Minhong Zhang, Junsong Ye

**Affiliations:** ^1^ Subcenter for Stem Cell Clinical Translation, First Affiliated Hospital of Gannan Medical University, Ganzhou, Jiangxi, China; ^2^ School of Rehabilitation Medicine Gannan Medical University, GanZhou City, Jiangxi, China; ^3^ The First Clinical College of Gannan Medical University, Ganzhou, Jiangxi, China; ^4^ Clinical Medicine Research Center, First Affiliated Hospital of Gannan Medical University, Ganzhou, Jiangxi, China; ^5^ Key Laboratory of Prevention and Treatment of Cardiovascular and Cerebrovascular Diseases, Ministry of Education, Gannan Medical University, Ganzhou, Jiangxi, China; ^6^ Key Laboratory of Biomaterials and Biofabrication in Tissue Engineering of Jiangxi Province, Gannan Medical University, Ganzhou, Jiangxi, China; ^7^ Ganzhou Key Laboratory of Stem Cell and Regenerative Medicine, First Affiliated Hospital of Gannan Medical University, Ganzhou, Jiangxi, China

**Keywords:** cerebral palsy, stem cell therapy, meta-analysis, efficacy, safety

## Abstract

**Aim:** Although the efficacy and safety of stem cell therapy for cerebral palsy has been demonstrated in previous studies, the number of studies is limited and the treatment protocols of these studies lack consistency. Therefore, we included all relevant studies to date to explore factors that might influence the effectiveness of treatment based on the determination of safety and efficacy.

**Methods:** The data source includes PubMed/Medline, Web of Science, EMBASE, Cochrane Library, from inception to 2 January 2022. Literature was screened according to the PICOS principle, followed by literature quality evaluation to assess the risk of bias. Finally, the outcome indicators of each study were extracted for combined analysis.

**Results:** 9 studies were included in the current analysis. The results of the pooled analysis showed that the improvements in both primary and secondary indicators except for Bayley Scales of Infant and Toddler Development were more skewed towards stem cell therapy than the control group. In the subgroup analysis, the results showed that stem cell therapy significantly increased Gross Motor Function Measure (GMFM) scores of 3, 6, and 12 months. Besides, improvements in GMFM scores were more skewed toward umbilical cord mesenchymal stem cells, low dose, and intrathecal injection. Importantly, there was no significant difference in the adverse events (RR = 1.13; 95% CI = [0.90, 1.42]) between the stem cell group and the control group.

**Conclusion:** The results suggested that stem cell therapy for cerebral palsy was safe and effective. Although the subgroup analysis results presented guiding significance in the selection of clinical protocols for stem cell therapy, high-quality RCTs validations are still needed.

## 1 Introduction

Cerebral palsy (CP) was first described as cerebral paresis by Little in 1861 (Little, 1861). The appropriate definition of CP is difficult owing to the heterogeneity of the diseases (Colver et al., 2014). In 2005, CP has been defined as a group of disorders of the development of movement and posture that cause activity limitation by the Executive Committee for the Definition of CP (Bax et al., 20052005). Specifically, CP is attributed to a non-progressive disturbance that occurred in the developing fetal or infant brain (Chin et al., 2022). The phenotypic motor disorders of CP are often accompanied by disturbances of sensation (Greene, 2021), cognition (Stadskleiv, 2020), communication (Mei et al., 2020), perception (Aisen et al., 2011) and epilepsy (El Tantawi et al., 2019), which generates great pain to both the patient and the family. The pooled overall prevalence of CP was 2.11 per 1,000 live births (Oskoui et al., 2013) all over the world, and the pooled prevalence of CP over the 32 years from 1988 to 2020 was 2.07‰ in China (Yang et al., 2021). Meanwhile, CP exerted higher prevalence in low- and middle-income countries than that in high-income countries. For example, the objective observed prevalence was 3.4 per 1,000 children in Bangladesh (Khandaker et al., 2019).

Currently, it still remains unclear whether the medical drugs, surgery or rehabilitation means merely aiming to reduce secondary musculoskeletal deformity in CP, rather than treat the primary central neurological deficit (Colver et al., 2014). Whereas, compared with traditional chemical drugs, stem cells as “drugs” are characterized by non-targeting, multi-potential and flexible function, which makes them have the potential to treat complicated diseases. For example, stem cells have several functions that might be critical to the treatment of CP including immune regulation (Bennet et al., 2012), paracrine effects (Lv et al., 2021), angiogenesis (Kiasatdolatabadi et al., 2017), and neuroplasticity (Jantzie et al., 2018). Collectively, stem cell transplantation is considered a promising therapeutic strategy in clinical practice (Xie et al., 2020), and the effectiveness of stem cell transplantation in the treatment of CP has been preliminarily verified by evidence-based medicine (Novak et al., 2016; Eggenberger et al., 2019; Xie et al., 2020; Smith et al., 2021). However, we found that stem cell treatment protocols were non-uniform across studies, and previous systematic reviews did not provide appropriate recommendations on factors that may affect the therapeutic effect, such as cell type selection, dose and administration. Herein, we aim to rigorously screen and extract all clinical trial data on stem cell therapy (SCT) for CP, and objectively evaluate and summarize evidence of SCT for CP symptoms through systematic review and meta-analysis. In addition, based on the results of subgroup analysis, we also provide suggestions for the selection of treatment options, in order to promote the clinical application of SCT in CP.

## 2 Methods

The detailed protocol is registered in the PROSPERO (CRD42022301070, https://www.crd.york.ac.uk/PROSPERO/). The preferred reporting checklist (PRISMA) of systematic reviews and meta-analysis were used to guide this study (Supplementary Material S1).

### 2.1 Inclusion criteria

1). Population: patients diagnosed with CP, regardless of region, gender or race; 2). Intervention: stem cells therapy in combination with or without other treatments; 3). Comparisons: rehabilitation therapy and regular medication; d). Outcomes: the indicators are the scores of Gross Motor Function Measure (GMFM), Comprehensive Function Assessment (CFA), Gross Motor Performance Measure (GMPM), Bayley Scales of Infant and Toddler Development (BSID-Ⅱ) and Functional Independence Measure for Children (WeeFIM) or any other evaluation tools suitable for CP; e). Study Types: Randomized Controlled Trials (RCTs) that paralleled or crossover.

### 2.2 Exclusion criteria

Reports, reviews, abstracts, trials and letters with duplicate, incomplete and unavailable data were excluded. In addition, studies that are not relevant to the topic of this paper (such as studies using animal models or *in vitro* models as experimental subjects and using interventions that are not stem cell transfusions) are excluded.

### 2.3 Data sources

The following English databases were searched from the inceptions to 2 January 2022: PubMed/Medline, Web of Science, EMBASE, Cochrane library. The MeSH and keywords search terms included Stem Cells, Progenitor Cells, Mother Cells, CP, Dystonic-Rigid and Cerebral Palsies. A detailed illustration of search strategies is available in Supplementary Material S2.

### 2.4 Data extraction and quality assessment

Two independent reviewers evaluated the retrieved studies for inclusion and assessed the methodological quality of included studies. Elements extracted included study characteristics (author, country, publication year and design), participant characteristics (sex, age range and diagnostic criteria), intervention details (types of cells, dose ranges, administration and frequency), outcome measures, and follow-up time. The risk of bias was assessed using ROB2 (Risk of bias tool 2) (Higgins et al., 2011). The disagreements were thrashed out by the additional reviewer.

### 2.5 Data analysis

Data entry and analysis were performed using Review Manager 5.3 software. The data required for meta-analysis was directly extracted from the original literature or indirectly calculated on the basis of the original data through the conversion tool (https://www.yxzlb.com/forum.php?mod=viewthread&tid=3679&page=1#pid9919) developed by Chinese scholars (For example, SE of GMFM in study Kang et al. (2015) needs to be converted into standard deviation (SD), and SD of GMFM in study (Rah et al., 2017) needs to be calculated by *p*-value and sample size.). Since CFA, GMPM and WeeFIM were used uniformly in various studies, the fixed-effect model and its index WMD were used in their combined analysis. The random effects model and its indicator, SMD, were used in the combined analysis of GMFM and BSID-Ⅱ because the different versions of these scales used in the included studies resulted in large differences in the means. The weighted mean difference (WMD) and standardized mean difference (SMD) were used to compare continuous variables (GMFM, CFA, BSID-Ⅱ and GMPM), while risk ratio (RR) was used to compare binary variables (Adverse events). All results obtained were reported with 95% confidence intervals (CI). Heterogeneity among studies was determined by Q test and I^2^ statistics [Cochrane book 9.5.2 Identifying and measuring heterogeneity, 0%–40%:might not be important; 30%–60%: may represent moderate heterogeneity*; 50%–90%: may represent substantial heterogeneity*; 75%–100%: considerable heterogeneity* (Cumpston et al., 2019)]. With substantial heterogeneity, sensitivity analysis or subgroup analysis was used to detect the source of heterogeneity; if the source of heterogeneity cannot be found, a descriptive analysis was conducted. Meanwhile, funnel plots were used to assess publication bias. For trials that had a crossover design, we included all the data before and after the crossover. When studies of multiple intervention groups are compared, the “shared” control group is split equally in each comparison.

## 3 Results

### 3.1 Results of the search

**FIGURE 1:**
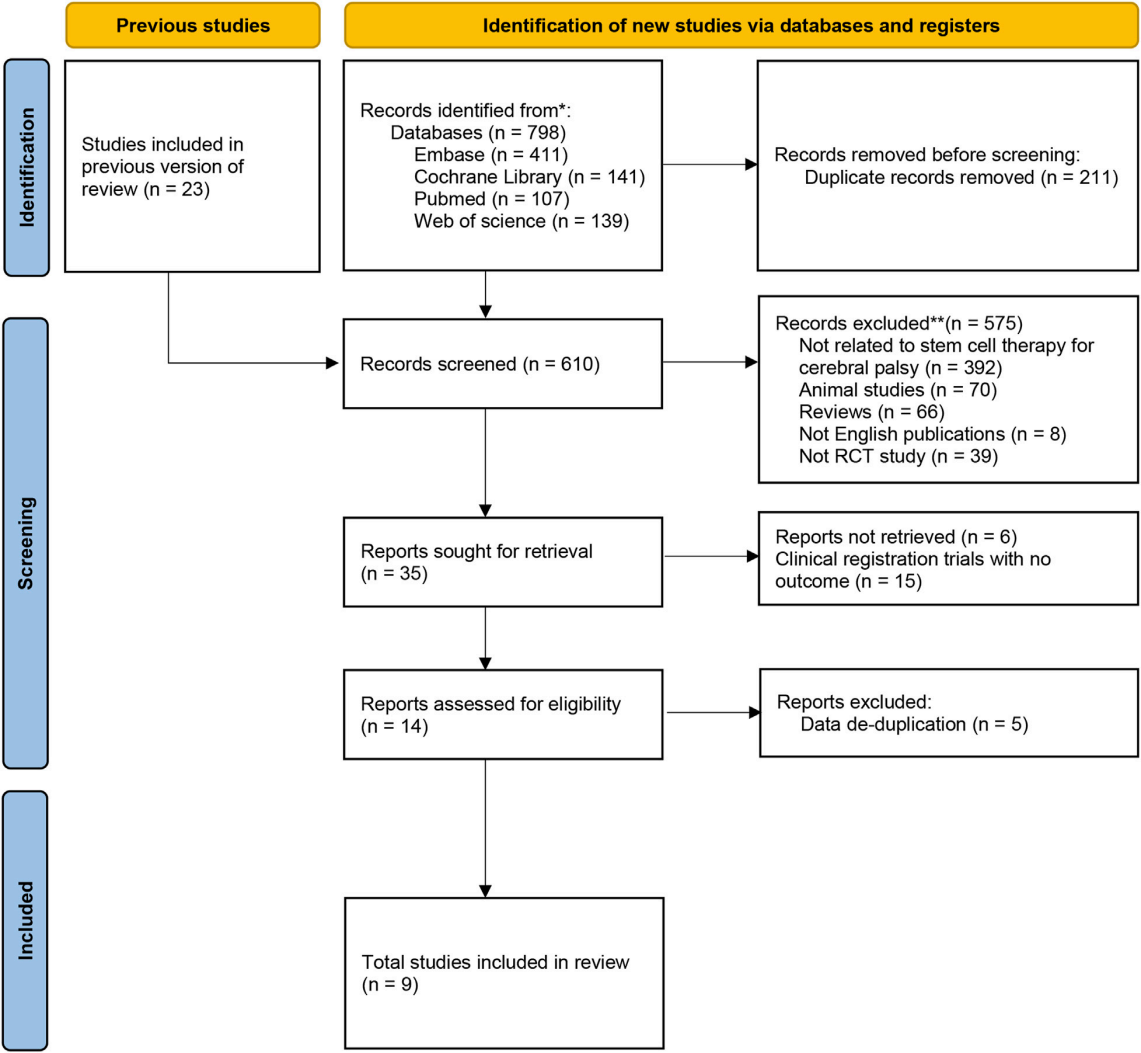
The inclusion flow chart of the literature was retrieved.

A flowchart describing the selection of eligible trials is presented in Figure 1. A total of 798 articles from 4 databases were retrieved: Web of Science (*n* = 139) databases, PubMed/MEDLINE (n = 107), Cochrane (*n* = 141), Embase (*n* = 411). Studies (*n* = 23) from previously published reviews (Kułak-Bejda et al., 2016; Novak et al., 2016; Eggenberger et al., 2019; Xie et al., 2020) were also included for screening. After reexamination and other screening, 35 studies were included. However, 15 of the studies were clinical registration trials with no outcome, 6 of the conference abstracts without full text and relevant data, and 5 of the studies were duplicated with data from other literatures. Finally, 9 studies were included in our meta-analysis.

### 3.2 Characteristics of the studies

The characteristics of the included studies are listed in Table 1. Two studies recruited patients from Iran and America respectively. Four of the remaining seven studies came from China and three from South Korea. Except for Huang et al. (2018) was single-blind RCT and Luan et al. (2012) did not report blindness, the rest were double-blind RCT designs, among which Sun et al. (2017) and Rah et al. (2017) were crossover designs study Liu et al. (2017) conducted simultaneous interventions of two stem cell types, bone marrow mesenchymal stem cell (BMMSC) and bone marrow mononuclear cell (BMMNC) on CP. Therefore, we divided them into two groups for data extraction.Amanat et al. (2021) and Liu et al. (2017) included only patients with spastic CP in their studies. All the studies’ sample sizes ranged from 36 to 105 and were published from 2012 to 2021. The main transfused routes are intravenous infusion and intrathecal injection, which total dose ranged from 4 × 10^6^–5.2 × 10^8^/kg.

**TABLE 1:** Summary of Clinical Studies of Stem Cells Therapy for cerebral palsy.

Study	Country	Design	Sample size	Age, y	Patient condition	Therapy				Control				Follow up	Main outcome measures
						Sample size	Average age, y	Cell type	Dose	Administration	Sample size	Average age, y	Control intervention		
Amanat et al. (2021)	Iran	RCT double-blind	72	4–14	spastic CP, GMFCS level 2–5, white matter lesions	36	8.475	hUC-MSC	2 × 10^7^/kg, single time	intrathecal route	36	8.542	sham procedure, Bobath therapy	1, 3, 6, and 12-month	GMFM-66, MAS, PEDI, CP-QoL, FA and MD of CST and PTR.
Gu et al. (2020)	China	RCT double-blind	40	2–12	CP	20	3.83	hUC-MSC	4.5–5.5 × 10^7^/kg, 50ml, 4 times	intravenous infusion	20	4.775	Placebo, Bobath therapy and conductive education	12-month	GMFM-88, ADL, CFA,^18^F-FDG-PET/CT
Huang et al. (2018)	China	RCT single-blind	54	3–12	CP	27	7.3	hUCB-MSC	5× 10^7^/kg, 4 times	intravenous infusions	27	7.5	normal saline, basic rehabilitation	3, 6, 12, 24-month	GMFM-88, CFA, Lab test, EEG, MRI
Kang et al. (2015)	Korea	RCT double-blind	36	0.5–15	CP	18	3.9	UCB	5.46× 10^7^/kg	intravenous or intra-arterial routes	18	3.775	placebo	2-week and 1, 3, 6-month	MMT, GMFM, GMPM, BSID-II, WeeFIM^®^, PEDI, 18F-FDG-PET
Liu et al. (2017)	China	RCT double-blind	105	0.5–12.5	spastic CP	35	4.129	A: BMMSC	1 × 10^6^/kg, four times	intrathecal injections	35	4.105	Bobath therapy	3, 6, and 12-month	GMFM, FMFM
						35	4.092	B: BMMNC							
Luan et al. (2012)	China	RCT	94	0.4–3.3	CP	49	1.083	NPCs	8–10 × 10^6^, 200 µL	lateral ventricles	45	1.569	rehabilitation training	1 year	GMFM, PDMS, Survey questionnaire
Min et al. (2013)	Korea	RCT double-blind	96	0.5–7.3	CP	31	3.067	UCB + RhEPO	TNCs ≥3 × 10^7^/kg	intravenous infusion	32	3.192	rehabilitation training	1, 3, 6-month	GMPM, BSID-II Mental and Motor scales, GMFM, WeeFIM, 18F-FDG-PET/CT
Rah et al. (2017)	Korea	RCT double-blind, crossover	47	2–10	CP	47	4.1	mPBMC	5.2 × 10^8^/kg	intravenous infusion	47	4.1	personalized physiotherapy and occupational therapies	6-month	GMFM, PEDI, QUEST
Sun et al. (2017)	America	RCT double-blind	63	1–6	CP	32	2.1	ACB	1–5×10^7^/kg	intravenous infusion	31	2.3	placebo, traditional rehabilitation therapies	1,2-year	PDMS-2, GMFM-66

Gross motor function measure (GMFM), modified Ashworth scale (MAS), pediatric evaluation of disability inventory (PEDI), CP quality of life (CP-QoL), fractional anisotropy (FA), Comprehensive Function Assessment (CFA), Gross Motor Performance Measure (GMPM), Functional Independence Measure for Children (WeeFIM), Bayley Scales of Infant and Toddler Development (BSID-Ⅱ), mean diffusivity (MD), corticospinal tract (CST), posterior thalamic radiation (PTR), human umbilical cord mesenchymal stem cells (hUC-MSC), human umbilical cord blood mesenchymal stem cell (hUCB-MSC), umbilical cord blood (UCB), Bone marrow mesenchymal stem cells (BMMSCs), bone marrow mononuclear cells (BMMNCs), neural progenitor cells (NPCs), Peabody Developmental Motor Scale-Fine Motor (PDMS-FM), recombinant human erythropoietin (rhEPO), Quality of Upper Extremity Skills Test (QUEST), Manual Ability Classification System (MACS), autologous cord blood (ACB), total nucleated cell (TNC)

### 3.3 Risk assessment of bias

**FIGURE 2:**
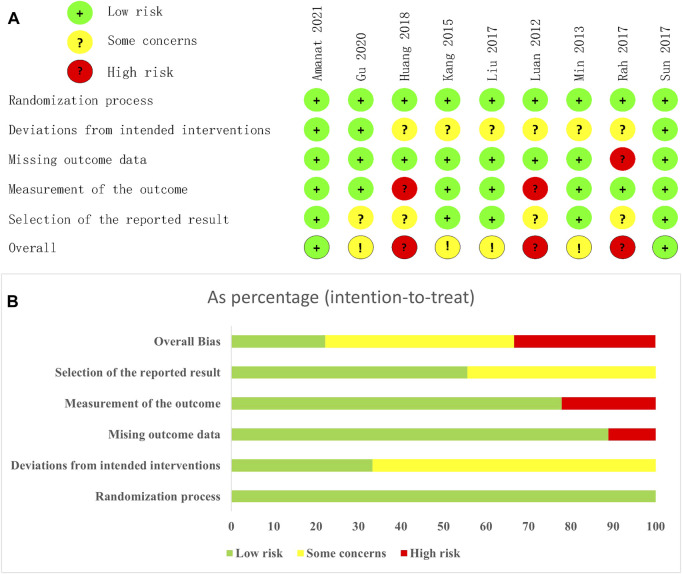
Risk of bias graph. **(A)** each risk of bias item for each included study. **(B)** each risk of bias item presented as percentages across all included studies.

Figure 2 showed the assessment results of bias risk and methodological suitability of the included studies. As all the 9 included studies were RCTs, the bias arising from the randomisation process was low risk. Only three studies used appropriate analyses to estimate the effect of assignment to intervention. In addition, Huang et al. (2018) was a single-blind design and Luan et al. (2012) was a non-blind design, which makes carers and people delivering the interventions aware of the participants’ assigned intervention during the trial. Therefore, bias due to deviations from intended intervention of 6 studies are considered some concerns. Bias due to missing outcome date was high risk in Rah et al. (2017), as the availability of date small than 95%. Huang et al. (2018) and Luan et al. (2012)’s bias in measurement of the outcome was high risk due to the implementation of the inappropriate blind method mentioned above. Besides, Gu et al. (2020) and Rah et al. (2017)’s clinical trial registration status was retrospectively registration, and Huang et al. (2018) and Luan et al. (2012) did not provide information on clinical registration. We believe that their bias in selection of the reported result were some concerns. Detailed results of the ROB2 assessment are provided in Supplementary Material S3.

### 3.4 Meta-analysis

Nine eligible articles were meta-analyzed using a random effects model, with GMFM as primary and CFA, GMPM, BSID-Ⅱ, WeeFIM as secondary indicators to evaluate the effectiveness of SCT for CP, and adverse events (AE) as a safety indicator.

#### 3.4.1 Primary indicators

**FIGURE 3:**
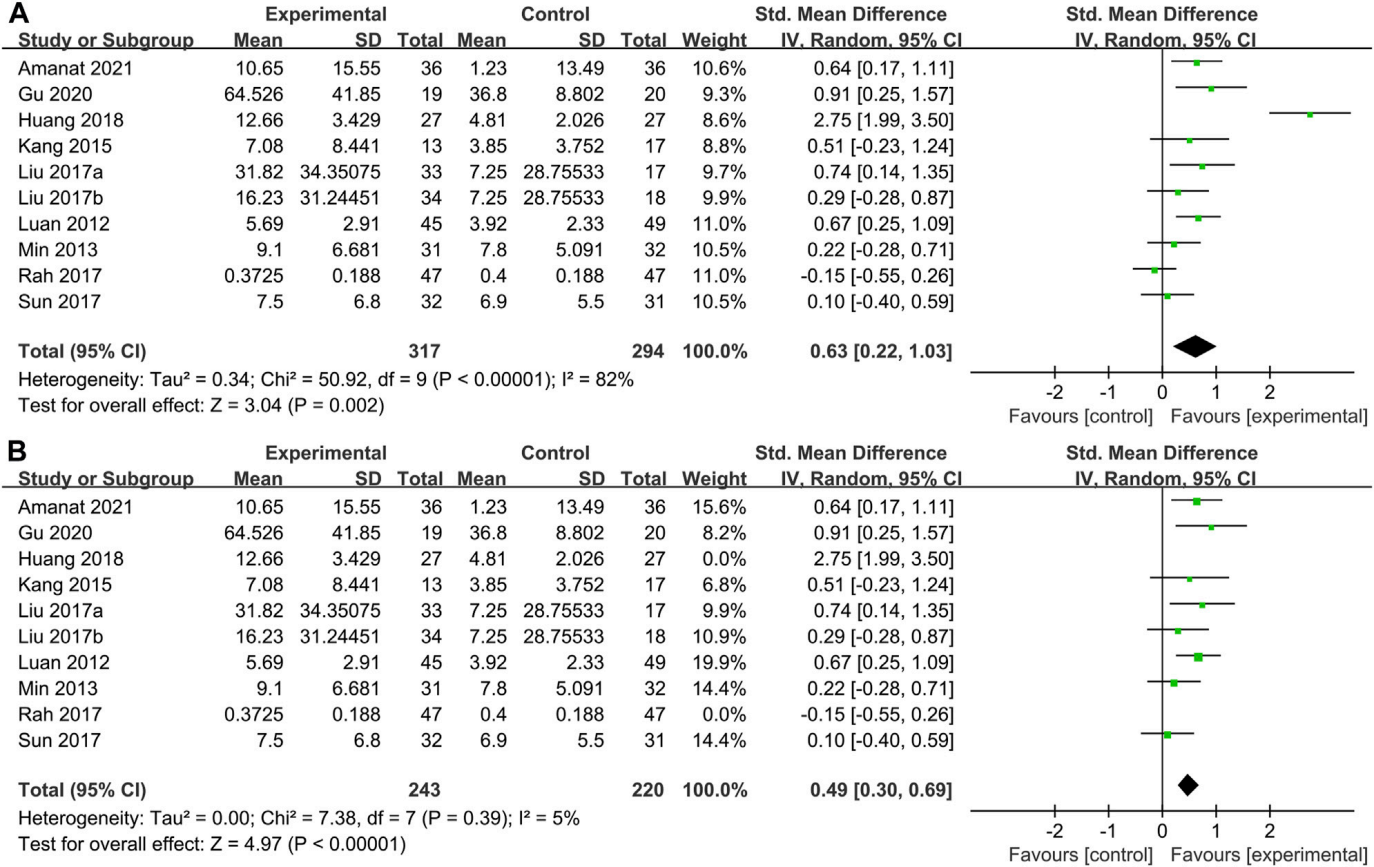
Primary indicators. Forest plot of GMFM. **(A)** Pooled results before sensitivity analysis. **(B)** Pooled results after eliminating heterogeneous sources.

GMFM scores were reported in 9 studies of 317 patients with SCT and 329 patients in the control group. We found that although every article used the GMFM score as one of the outcome indicators, some articles used GMFM-88 while others used GMFM-66, which is a simplified version of the former, leading to a huge difference in the mean value. So SMD was selected as the effect indicator. Data showed that the GMFM score of the stem cell group was significantly higher than the control group (SMD: 0.63; 95% CI [0.22, 1.03]; *p* = 0.002) (Figure 3A). A higher score of GMFM refers to the lighter symptoms. However, heterogeneity test *p* < 0.00001; I^2^ = 82%, indicating considerable heterogeneity. Sensitivity analysis suggested that Huang et al. (2018) and might be the source of heterogeneity, when they were removed, the results showed that the heterogeneity was greatly reduced (*p* = 0.39; I^2^ = 5%) and the results were more stable (SMD: 0.49; 95% CI [0.30, 0.69]; *p* < 0.00001) (Figure 3B). Before sensitivity analysis, funnel plot corresponding to forest map showed skewness distribution. After sensitivity analysis, the source of heterogeneity was removed and the funnel plot was normally distributed (Supplementary Material S4).

#### 3.4.2 Secondary indicators

**FIGURE 4:**
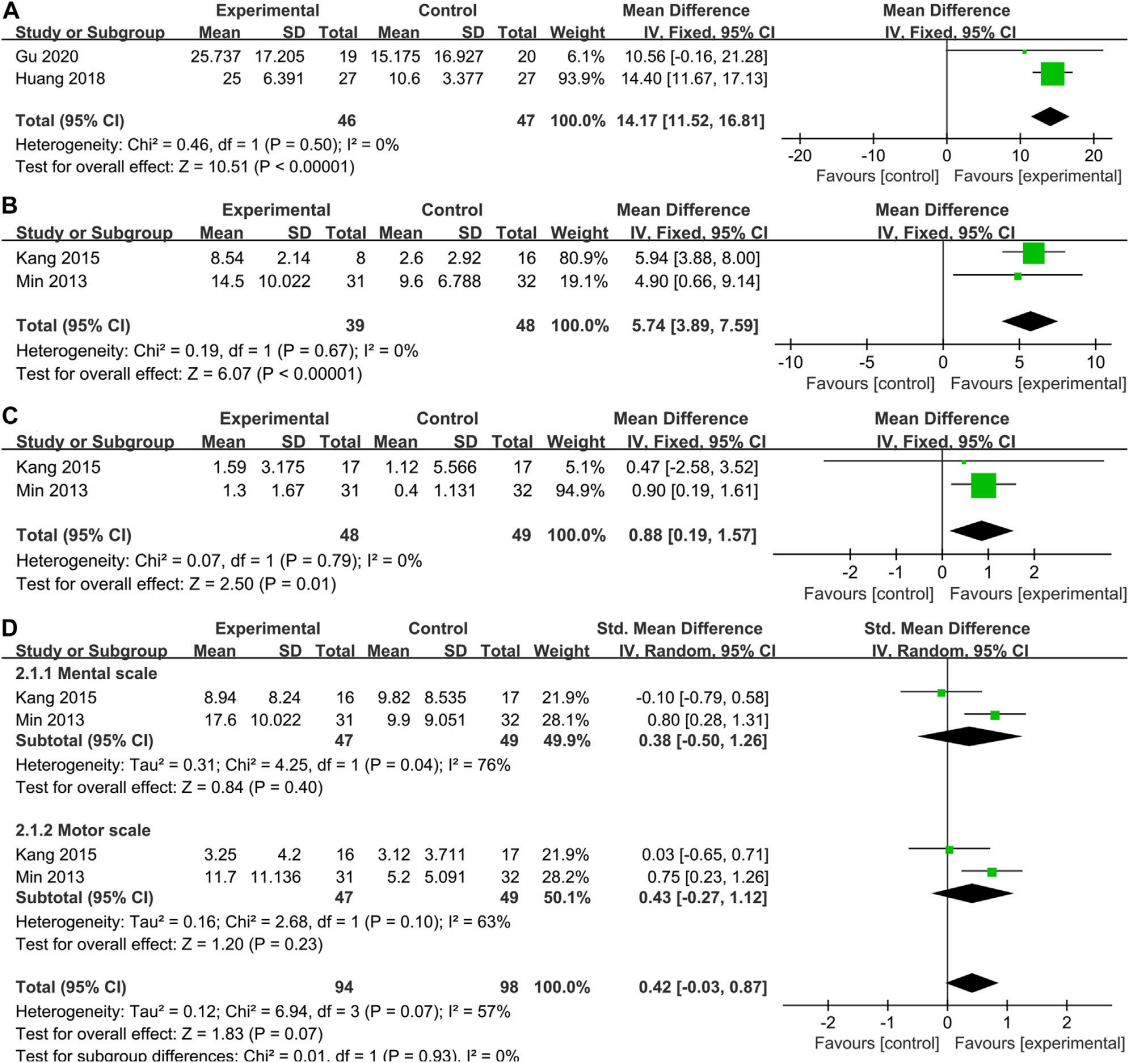
Secondary indicators. Forest plot demonstrating the effect of stem cells compared with controls on **(A)**CFA, **(B)** GMPM, **(C)** WeeFIM and **(D)** BSID-Ⅱ.

CFA scores were reported in 2 studies of 46 patients with SCT and 47 patients in the control group. From the meta-analysis (Figure 4A), stem cells greatly improved performance compared with controls on the CFA (WMD: 14.17; 95% CI: 11.52, 16.81; *p* < 0.00001; Heterogeneity test I^2^ = 0%, *p* = 0.50).

Our systematic literature review identified 2 studies (n = 87 participants) that investigated the effectiveness of SCT on GMPM in children with CP. Pooled analysis indicated that SCT significantly improved GMPM scores (WMD = 5.74, 95% CI = 3.89–7.59, *p* < 0.00001; Heterogeneity test I^2^ = 0%, *p* = 0.67) (Figure 4B), compared with the control group.

Two studies (Min et al., 2013; Kang et al., 2015) collected WeeFIM outcome data. Pooled analysis showed that the WeeFIM score of the stem cell group was significantly higher than the control group (WMD: 0.88; 95% CI [0.19, 1.57]; *p* = 0.01; Heterogeneity test I^2^ = 0%, *p* = 0.97) (Figure 4C).

In the two studies (Min et al., 2013; Kang et al., 2015), BSID-Ⅱ scores were reported as mental scale and motor scale, so we also conducted a subgroup analysis of the results of BSID-Ⅱ (Figure 4D). Regrettably, there was no difference in either mental scale (WMD = 0.38, 95% CI = -0.50–1.26, *p* = 0.40; Heterogeneity test I^2^ = 76%, *p* = 0.04) or motor scale (WMD = 0.43, 95% CI = -0.27–1.12, *p* = 0.23; Heterogeneity test I^2^ = 63%, *p* = 0.10) scores between the stem cell treatment group and the control group. However, it is worth noting that the heterogeneity in the pooling of the two parts of the scale is greatly high.

#### 3.4.3 Subgroup of gross motor function measure

##### 3.4.3.1 Time subgroup of gross motor function measure

**FIGURE 5:**
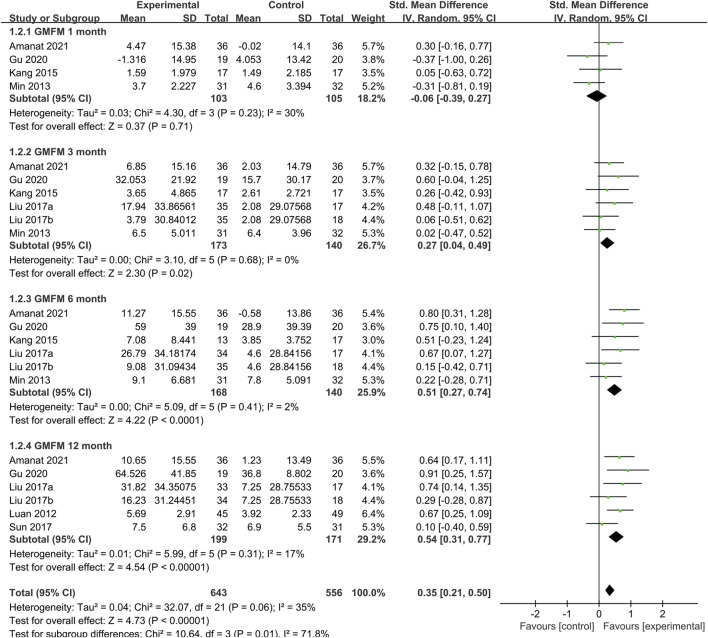
Time subgroup of GMFM.

At the same time as the treatment follow-up endpoint data were extracted, the follow-up node data for each study were also extracted. We performed the time subgroup analysis for GMFM, pooled analysis showed that SCT significantly increased GMFM scores (SMD = 0.35, 95%CI = [021, 0.50], *p* < 0:00,001, heterogeneity test *p* = 0.06; I^2^ = 35%) (Figure 5), compared with the control group. Subgroup analysis with random-effects model showed that SCT significantly increased GMFM scores in 3 months (SMD: 0.27; 95%CI [0.04, 0.49]; *p* = 0.02), 6 months (SMD: 0.51; 95%CI [0.27, 0.74]; *p* < 0.0001; heterogeneity test *p* = 0.41; I^2^ = 2%), and 12 months (SMD: 0.54; 95%CI [0.31, 0.77]; *p* < 0.00001; heterogeneity test *p* = 0.31; I^2^ = 17%.). Whereas, comparisons between the two groups showed no difference in 1 month (SMD = −0.06, 95%CI = −0.39–0.27, *p* = 0.71; heterogeneity test *p* = 0.23; I^2^ = 30%).

##### 3.4.3.2 Stem cells type subgroup of gross motor function measure

**FIGURE 6:**
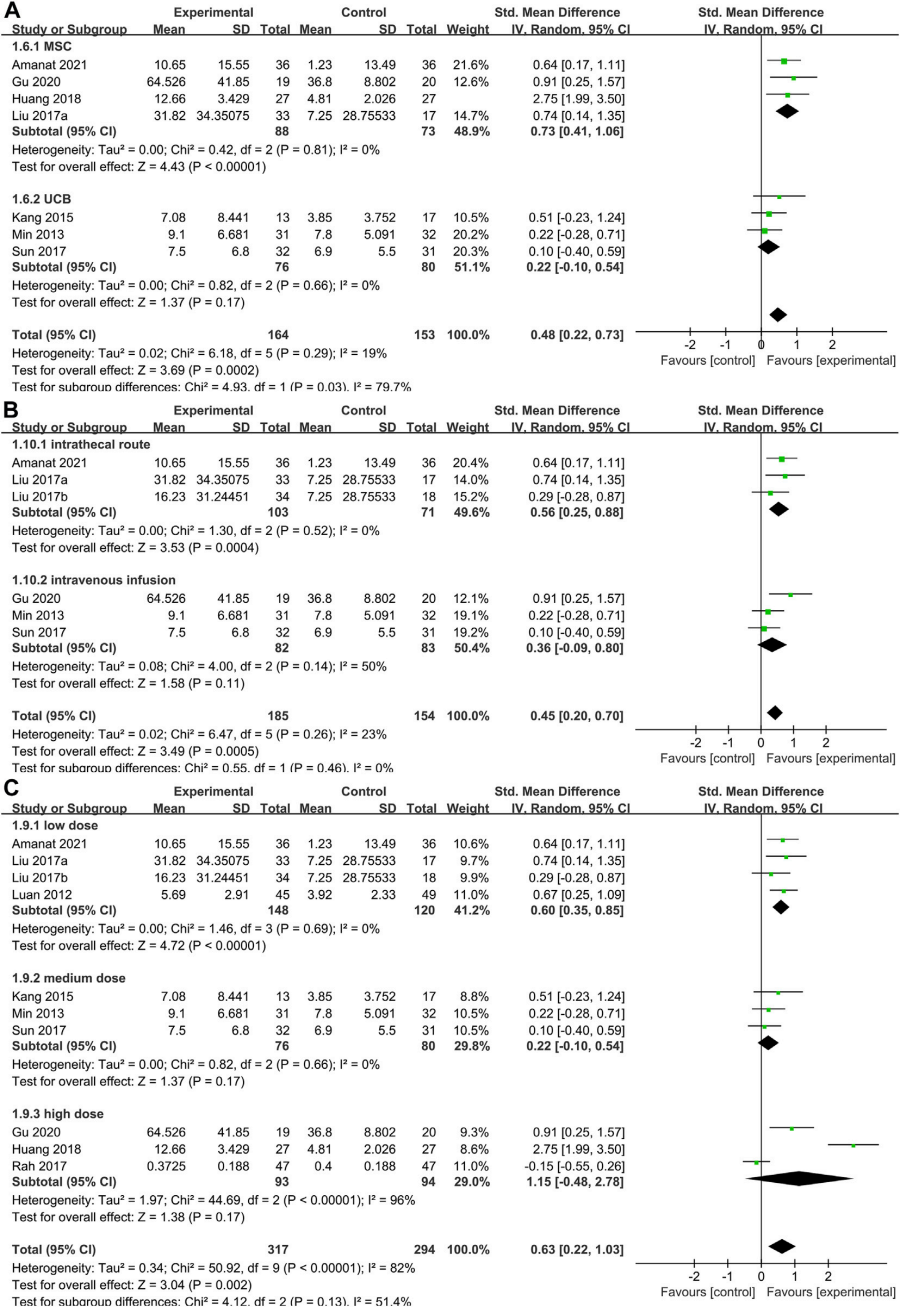
Forest plot of subgroup in GMFM. Forest plot demonstrating the effect of stem cells compared with controls in subgroup of **(A)** stem cells types, **(B)** administration route and **(C)** dose.

To determine the optimal cell type for SCT in CP, we conducted a subgroup analysis of the two main cell types included in the studies (Figure 6A). Data showed that the GMFM score of the treatment group was significantly higher than the control group in MSC group (SMD: 0.73; 95%CI [0.41, 1.06]; *p* < 0.00001; heterogeneity test *p* = 0.81; I^2^ = 0%) (Figure 6A). In contrast, the GMFM score of the treatment group showed no difference with control group in UCB group (SMD: 0.22; 95%CI [-0.10, 0.54]; *p* = 0.17; heterogeneity test *p* = 0.66; I^2^ = 0%). There was significant heterogeneity between the two cell types (heterogeneity test *p* = 0.03; I^2^ = 79.7%).

##### 3.4.3.3 Administration route subgroup of gross motor function measure

As mentioned above, the methods of cell transplantion in the included studies were mainly intravenous and intrathecal injection. Pooled analysis (Figure 6B) showed that SCT significantly increased GMFM scores (SMD = 0.45, 95%CI = 0.20–0.70, *p* = 0.0005), compared with the control group. Subgroup analysis with random-effects model showed that SCT significantly increased GMFM scores in intrathecal route (SMD = 0.56, 95%CI = 0.25–0.88, *p* = 0.0004; heterogeneity test *p* = 0.52; I^2^ = 0%). In contrast, no significant differences were observed in the intravenous subgroup (SMD = 036, 95%CI = -0.09–0.80, *p* = 0.11; heterogeneity test *p* = 0.14; I^2^ = 50%).

##### 3.4.3.4 Dose subgroup of gross motor function measure

Studies were divided into three grades based on the total number of cells injected: low dose (4×10^6^–3×10^7^/kg), medium-dose (3–9×10^7^/kg) and high-dose (9×10^7^–5.2×10^8^/kg). Pooled analysis (Figure 6C) showed that SCT significantly increased GMFM scores (SMD = 0.63, 95%CI = 0.22–1.03, *p* = 0.002), compared with the control group. Subgroup analysis with random-effects model showed that SCT significantly increased GMFM scores in low dose (SMD = 0.60, 95%CI = 0.35–0.85, *p* < 0.00001; heterogeneity test *p* = 0.69; I^2^ = 0%). However, the analysis results were not statistically significant at medium (SMD = 0.22, 95%CI = -0.10–0.54, *p* = 0.17) and high doses (SMD = 1.15, 95%CI = -0.48–2.78, *p* = 0.17).

##### 3.4.3.5 CP type subgroup of gross motor function measure

In the study of Amanat et al. (2021) and Liu et al. (2017) mentioned above, only patients with spastic CP were included, while other studies’ patients with CP were not classified. Therefore, we performed subgroup analysis on GMFM score for CP type (Supplementary Material S5A). The results suggested that SCT was effective in both spastic (SMD: 0.56; 95%CI [0.25, 0.88]; *p* = 0.0004; heterogeneity test *p* = 0.52; I^2^ = 0%) and unclassified CP (SMD: 0.45; 95%CI [0.16, 0.74]; *p* = 0.002; heterogeneity test *p* = 0.22; I^2^ = 31%) compared with the control group. But the difference between subgroups showed no statistically significance (*p* = 0.61, I^2^ = 0%).

##### 3.4.3.6 Age subgroup of gross motor function measure

The age range of patients included in the study was 0.5–15, and the mean age of the treatment groups ranged from 1 to 9 years in the 9 studies. Taking 4 years old as the dividing line, these studies were divided into two groups for subgroup analysis according to the average age of patients with stem cell infusion (Supplementary Material S5B). The results showed that GMFM improved significantly after SCT in the 1–4 years (SMD: 0.45; 95%CI [0.16, 0.74]; *p* = 0.002; heterogeneity test *p* = 0.22; I^2^ = 31%) and 4–9 years group (SMD: 0.56; 95%CI [0.25, 0.88]; *p* = 0.0004; heterogeneity test *p* = 0.52; I^2^ = 0%). There was no significant difference between the two subgroups (heterogeneity test *p* = 0.61; I^2^ = 0%).

#### 3.4.4 Safety indicator

**FIGURE 7:**
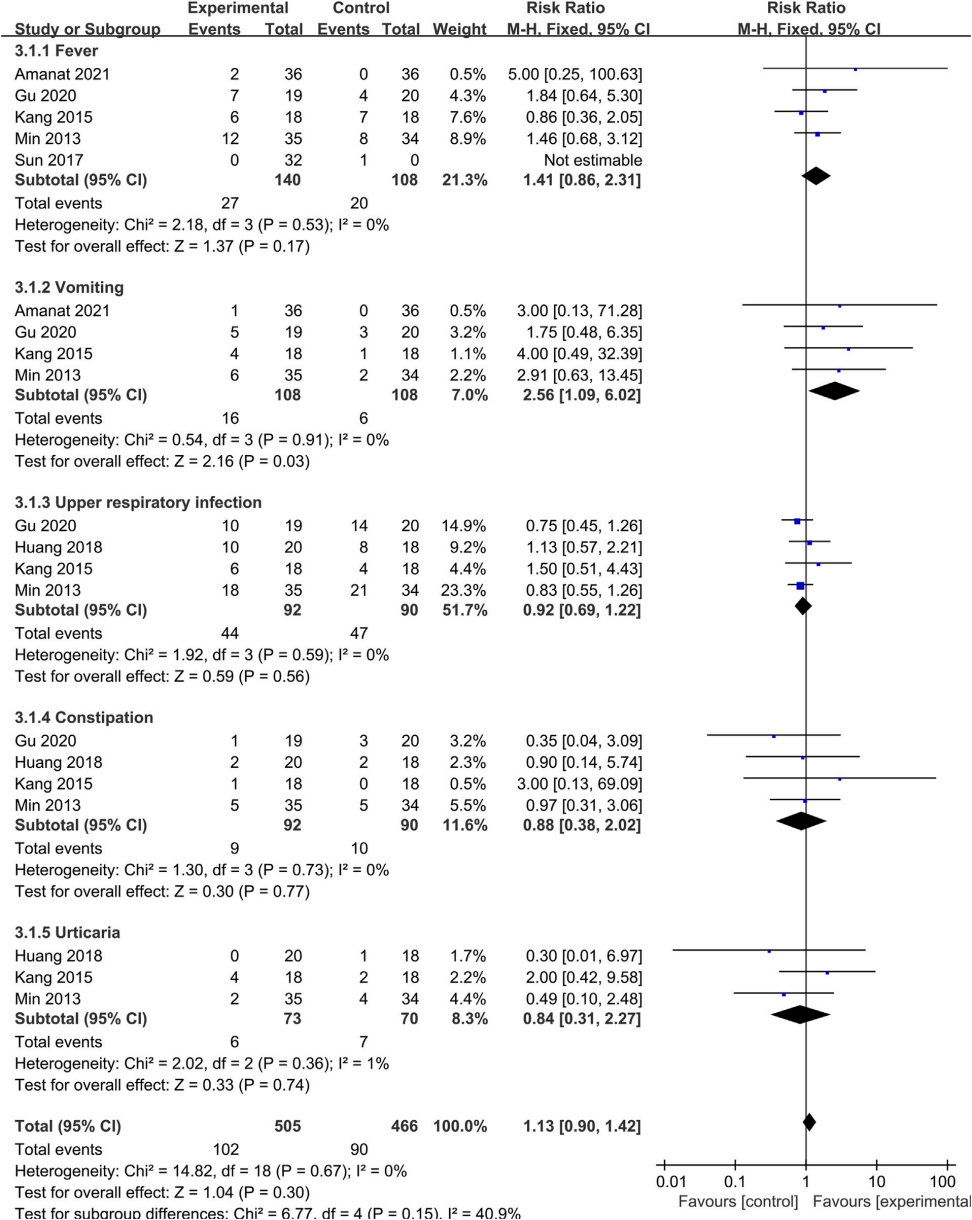
Forest plot of adverse events.

To explore the safety of SCT, we conducted a meta-analysis of AE. Studies that did not report AE in the control group were not included in this analysis. The number of AE of different types was reported in each study, and the total event frequency was larger than the sample size, which made direct data consolidation impractical. We performed a subgroup analysis of the same events (fever, vomiting, upper respiratory infection, constipation and urticaria) reported in each study to assess safety (Figure 7). Pooled analysis indicated that the overall effect was not statistically significant, and there was no difference in the incidence of AE between SCT and the control group (RR = 1.13; 95% CI = [0.90, 1.42]; *p* = 0.30; heterogeneity test *p* = 0.67; I^2^ = 0%). Although there was difference in the vomiting group (RR = 2.56; 95% CI = [1.09, 6.02]; *p* = 0.03; heterogeneity test *p* = 0.91; I^2^ = 0%), all studies indicated that there were no adverse consequences after symptomatic treatment or spontaneous remission.

### 3.5 Descriptive analysis

#### 3.5.1 magnetic resonance imaging and diffusion tensor imaging

Amanat et al. (2021) and Huang et al. (2018) suggested no significant improvements in the Magnetic Resonance Imaging (MRI) of participants were observed compared to the baseline. However, the Diffusion Tensor Imaging (DTI) analysis showed that mean fractional anisotropy (FA) increased significantly in the SCT group and was statistically higher than the control group according to two studies (Min et al., 2013; Amanat et al., 2021). In addition, Amanat et al. (2021) suggested the mean diffusivity (MD) decreased significantly in the experimental group and was statistically lower than the control group. And Min et al. (2013) revealed changes in FA of the spinothalamic tract in the right posterior lower pons, with the umbilical cord blood (UCB) group showing greater increments than did the other groups. On the contrary, in the MRI-DTI scans of (Rah et al., 2017), although there was a trend of increasing FA values and decreasing apparent diffusion coefficient (ADC) values over time, these trends were not statistically significant.

Sun et al. (2017) performed whole brain connectome analysis based on MRI diffusion-weighted images from all directions, which suggested patients who received total nucleated cell count (TNCC) > 2 × 10^7^/kg demonstrated a statistically significant greater increase in normalized whole brain connectivity 1 year after treatment than children who received lower doses. In the sensorimotor network, nodes with significant increases in connectivity that correlated with improvement in GMFM-66 scores included the pre- and post-central gyri, basal ganglia, and brain stem.

#### 3.5.2 Positron emission tomography/computed tomography

Both Gu et al. (2020) and Kang et al. (2015) showed significant improvements in brain activity. Interestingly, Kang et al. (2015) observed in the UCB group, increased activity in multiple cortical areas of the frontal and parietal lobes was accompanied by a significant decrease in bilateral white matter activity of the occipital and temporal lobes. The opposite was true for the control group. The results of (Rah et al., 2017) were consistent with the trend of DTI detection, although they observed metabolic changes to the cerebellum, thalamus and cerebral cortex in the brain PET-CT, there were no significant differences in the incidence of metabolic changes between the mobilized peripheral blood mononuclear cells (mPBMC) and placebo groups.

#### 3.5.3 Biochemical parameters

Kang et al. (2015) examined the patient’s biochemical parameters and performed a regression analysis with the GMFM score, suggesting a meaningful perspective that increases in PTX3 from baseline to 1-day post-treatment were correlated with improvements in GMPM at 1-month post-treatment, increases in IL-8 level from baseline to 12 days post-treatment were correlated with improvements in GMFM at 6 months post-treatment.

## 4 Discussion

There are limited treatments available for CP, and stem cells show promising therapeutic potential, with mounting clinical studies data. From 9 studies identified, this meta-analysis showed that stem cell administration significantly improved motor outcomes (GMFM, CFA, GMPM and WeeFIM). Besides, there was no statistical difference in the incidence of AE between the stem cell treatment group and the control group, which suggested that CP is safe to be treated with stem cells. Although SCT showed favorable results for CP patients, we can glimpse from the included studies where future CP stem cell therapies still need to be explored.

In clinical trials of Cochrane’s (www.cochranelibrary.com) registered stem cell treatment for CP (Supplementary Material S6), the cells used frequently were derived from the umbilical cord and bone marrow, and trials using umbilical cord blood mesenchymal stem cell (UCMSC) as an intervention were the most numerous. This may be related to the availability of umbilical cord and the low immunogenicity (Chamberlain et al., 2007) of MSC. Comparatively, these factors are only a part of the selection of treatment that should be considered, more important is the treatment efficacy. Subgroup analysis of GMFM showed that MSC had a more significant therapeutic effect on CP compared with UBC, which may guide the selection of stem cells for clinical application of SCT in CP. Nevertheless, there are still many sources of MSC, and it is not known whether this might affect the efficacy or not. In addition, there are studies on the differentiation of MSC into neural progenitor cells *in vitro* and infusion therapy (Luan et al., 2012), which also provides a new direction for SCT. Therefore, a large number of experiments are still needed to screen specific cell types for CP therapy.

Excitingly, a new generation of stem cell therapies, including Exosomes and genome-edited stem cells, is emerging, which may make the potential of stem cell-based therapies more apparent. Exosomes, a signaling molecule, have particular advantages as a new therapy. It not only has the same function as stem cells, but also has a more stable membrane structure than stem cells. Compared with whole-cell therapy, exosomes are well tolerated and have low immunogenicity (Tang et al., 2021). Using gene therapy and gene editing technology, it is possible to create more functional, specific and reactive stem cell derivatives based on traditional stem cells. For example, stem cells that overexpress neurotrophic factors, anti-inflammatory cytokines, or angiogenic factors can promote the healing and recovery of tissues damaged by injury or disease (Kimbrel and Lanza, 2020). In addition, the application of biomaterials in stem cell therapies can create spaces for cells to contact host tissues, establish platforms for delivery of various growth factors and seed cells, and provide better microenvironments for surviving cells (Li et al., 2022). These could make stem cell-based therapies more promising and offer more treatment options for complex neurological diseases.

The etiology of CP is heterogeneous (Jantzie et al., 2018; Shariati et al., 2021), resulting in children with CP having different symptoms. There are many classifications of CP, the most commonly used being the one proposed by Ingram in 1955 (Ingram, 1955), which includes the following clinical types: hemiplegia, double hemiplegia, diplegia, ataxia diplegia, Ataxia, and dyskinesia. It is necessary to identify the type of CP that best responds to SCT. We performed a subgroup analysis of studies in which all patients had spastic CP and studies in which subjects were composed of multiple CP types. The results showed that SCT improved GMFM scores in both subgroups, and since there was no difference between groups (subgroup differences *p* = 0.61; I^2^ = 0%), we could not identify which type of CP that was more sensitive to SCT.

Most children with CP are accompanied by one or more of the following symptoms (Novak et al., 2012): pain, intellectual disability, walking difficulty, dysphonia, epilepsy, bladder control problems, sleep disorder, blind or dysphagia. These secondary symptoms may worsen over time, limiting the effectiveness of treatment. Therefore, in CP rehabilitation, early intervention is important to optimize infant motor and cognitive plasticity (Novak et al., 2017). So, is it also better to inject stem cells sooner rather than later? Rosenblum et al. (2012) suggested that three days after hypoxia-ischemia in mice was the optimal time for NSC arterial transplantation. Obviously and regrettably, it is not clinically possible to achieve such rapid diagnosis and cell transplantation. We divided the study into two subgroups of 1–4 and 4–9 years old according to age. The results showed that there was no difference between the two groups, which may be related to the small age range included in the study. Nevertheless, the effect of age at intervention on the outcome of SCT should be determined before SCT is widely used.

Subgroup analysis with a random-effects model showed that SCT significantly increased GMFM scores in 3, 6 and 12 months, compared with the control group in CP (Figure 5). However, GMFM scores exhibited no differences at 1 month. This may be related to the limitations of the evaluation tools. Hielkema (Hielkema et al., 2013) suggested the GMFM-66 differentiated less at lower-ability levels than at higher-ability levels. The GMFM-88 demonstrated flattening of the developmental curve when infants had developed more motor abilities. Longitudinal use of the GMFM in infancy was hampered by age and function-specific limitations. This may suggest that more sensitive to post-treatment assessment of CP need to be developed based on different developmental stages of CP patients.

Stem cell plays the therapeutic role mainly through the direct differentiation into target cells under a specific microenvironment, subsequently, replacing the damaged or missing cells (Maroof et al., 2013). Meanwhile, exogenous stem cells could migrate to the damaged site and activate endogenous stem cells *in situ* differentiating into target cells along with regulating the niche by paracrine secretion (Siniscalco et al., 2013; Segal-Gavish et al., 2016; Perets et al., 2017). The migration and homing of stem cells are influenced by multiple factors such as number of cells and administration (Sohni and Verfaillie, 2013). Unfortunately, there is no standard dose and delivery method protocol for SCT on CP. In included studies, the dose of cell infusion varied, with the lowest being 4×10^6^/kg and the highest being 5.2 × 10^8^/kg. Sun et al. (2017) observed no difference in GMFM-66 scores between the UCB and placebo groups. However, his exploratory analysis showed that subjects who received a TNCC >2 × 10^7^/kg improved a median of 4.3 points greater than expected, and this change was statistically different from that observed in subjects who received <2 × 10^7^/kg or placebo. Besides, Kang et al. (2015) showed participants who received cells more than the 5.46 × 10^7^/kg showed greater improvements in BSID-II motor raw score than those who received less cells. The two researchers’ data seem to suggest that the higher the dose, the better the treatment. Surprisingly, we expanded the dose range by combining the included studies, subsequent subgroup analysis showed that SCT significantly increased GMFM scores in low dose (4×10^6^–3 × 10^7^/kg) (SMD = 0.60, 95% CI = 0.35–0.85, *p* < 0:00,001), while the medium-dose (3–9 × 10^7^/kg) and high-dose (9 × 10^7^–5.2 × 10^8^/kg) groups did not differ from the control group. This suggested that higher cell doses did not confer the desired therapeutic effect. In addition, it has been reported that there is a risk of cell clumping resulting in embolism at high cell doses (Heng et al., 2008). Therefore, in order to achieve the best therapeutic effect, the optimal dose range needs to be determined, and our current conclusion is 4 × 10^6^–3 × 10^7^/kg. On the other hand, subgroup analysis of administration route showed that GMFM score improvement was more significant in the intrathecal injection group, while there was no significant difference between the intravenous infusion group and the control group. Intravenous infusion of cells limits therapeutic effects, as cells might be trapped in organs such as the lung, liver, or kidney after infusion, reducing the number of cells that homing a specific site (Peng et al., 2020; Nguyen et al., 2021). Nevertheless, our conclusions are based on the treatment outcomes of different stem cells, so more double-blind randomized controlled trials with cell dose and administration as independent variables should be needed in the future.

The imaging tests described above may be a new direction in the assessment of CP, which can be more sensitive to the improved activity of brain regions at the root of CP, rather than just changes in motor function. Even though Amanat et al. (2021) and Huang et al. (2018) did not observe changes in patients’ brains in MRI, their study and Min et al. (2013) showed improvement in FA using DTI technology. Gu et al. (2020). and Kang et al. (2015) observed improvements in metabolic activity of patients’ brains using PET-CT. It should not be ignored that long-term repeated imaging examinations may bring harm to patients with CP. Therefore, it is worth trying as Huang et al. (2018) links blood biochemical indicators with the prognosis of CP, but the results still need to be verified by a large number of studies. Early brain injury impacts concomitantly on motor and cognitive development and function (Hielkema and Hadders-Algra, 2016), yet few studies describe the cognitive functioning in this population. Further, cognitive impacts may be realized only later in childhood due to the protracted nature of cognitive development, relative to motor skill development (Hoare et al., 2018). Although the motor function related scale score of the stem cell treatment group was higher than the control group, there was no difference in the BSID score of the two groups, which may indicate that SCT may have a limited therapeutic effect on cognition, but the results should be interpreted with caution due to high heterogeneity.

Although we evaluated the safety and efficacy of SCT for CP and discussed the details that need to be improved in the treatment regimen, the study still had limitations. We evaluated and analyzed the heterogeneity of included outcomes and found that there was a high heterogeneity in GMFM scores. Sensitivity analysis indicated that the studies of Huang et al. (2018) and Rah et al. (2017) resulted in high heterogeneity. The heterogeneity of former may be caused by the data processing method which analyzed the scores at each follow-up stage accounted for the total score of the scale. The total number of Rah et al. (2017) participants was 57, but only 47 were involved in outcome analysis. The lack of available data may have led to the bias of the results, resulting in heterogeneity. Thankfully, if we exclude the two study, heterogeneity will return to I^2^ = 5% and the pooled results are consistent with the previous trend. In this paper, subgroup analysis was performed to discuss the factors that may influence the SCT for CP, such as cell type, dose, route of administration, type of CP, time point of follow-up, and age of intervention. Although the subgroup analysis yielded preliminary results, we believe that the conclusions based on GFMF alone are one-sided, and the assessment of CP should be comprehensive. In addition, only 9 literatures were included in this study, which makes the results of combined analysis may be biased. Therefore, high-quality RCTs are still necessary in the future to obtain more accurate results.

## 5 Conclusion

**FIGURE 8:**
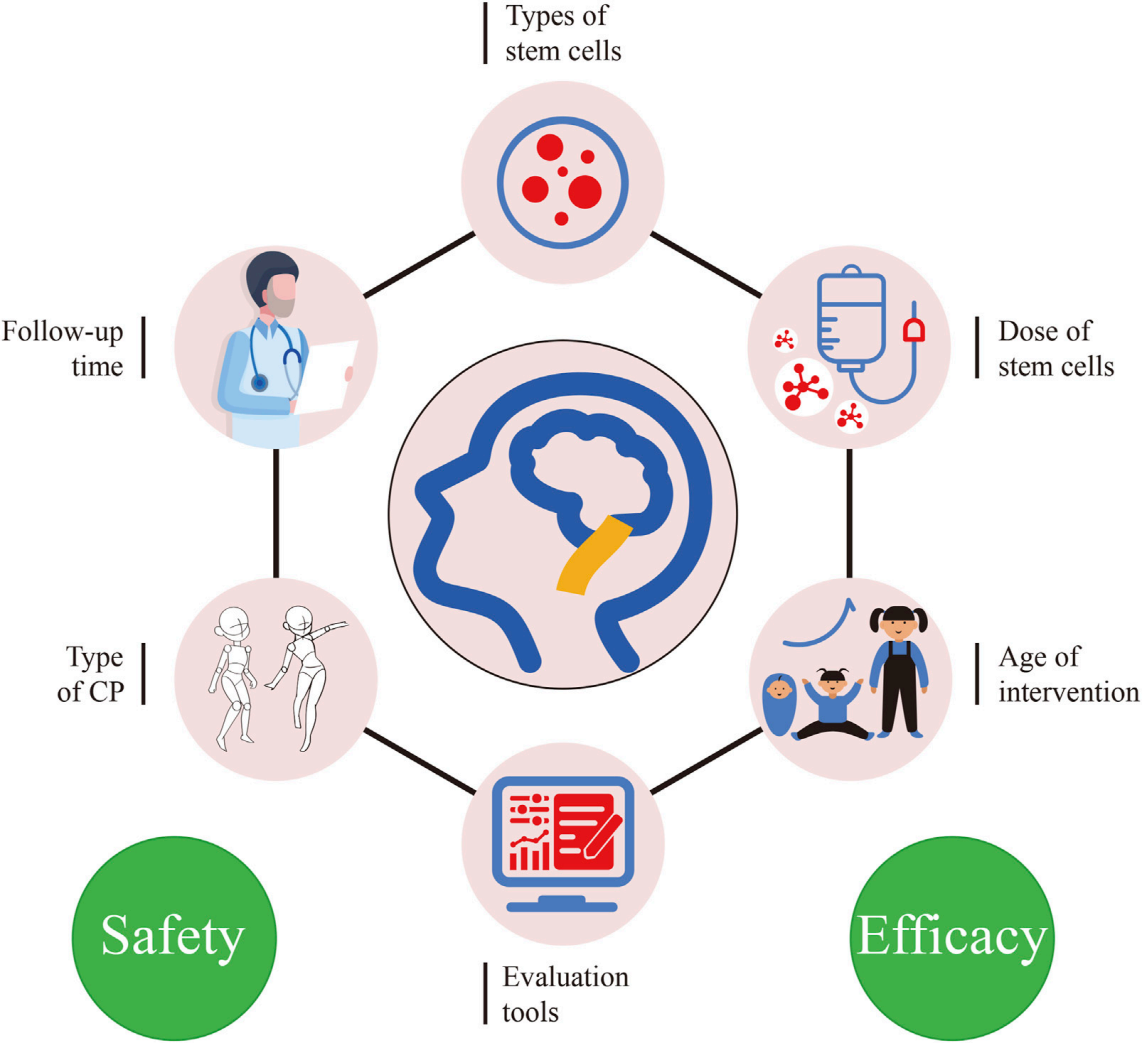
Factors that may influence stem cell therapy for CP.

In conclusion, the treatment of stem cells for CP was effective and safe, but the current treatment regimen is still not perfect. We summarize the factors that may influence the outcome of treatment in Figure 8. It is urgent to establish a standardized treatment protocol through a large number of trials, such as the most suitable stem cell type, dose and age of intervention need to be screened. These may lead to the discovery of SCT for CP and its pathogenesis, thus further improving the therapeutic effect. We expect SCT to be used in the clinical treatment of CP and have significant therapeutic effects, nevertheless, rehabilitation training is still essential. SCT improves the patient’s pathology, but rehabilitation therapy can accelerate the recovery of the patient’s limb function and social skills. In the future, SCT combined with rehabilitation therapy may be a new direction in the treatment of CP.

## Data Availability

The original contributions presented in the study are included in the article/Supplementary Material, further inquiries can be directed to the corresponding author.
