# Supplementary figures and images for: Efficacy and safety of stem cell therapy in cerebral palsy: A systematic review and meta-analysis
Source: Front Bioeng Biotechnol. 2022 Dec 14;10:1006845. doi: 10.3389/fbioe.2022.1006845 (PMC9794999; doi:10.3389/fbioe.2022.1006845)

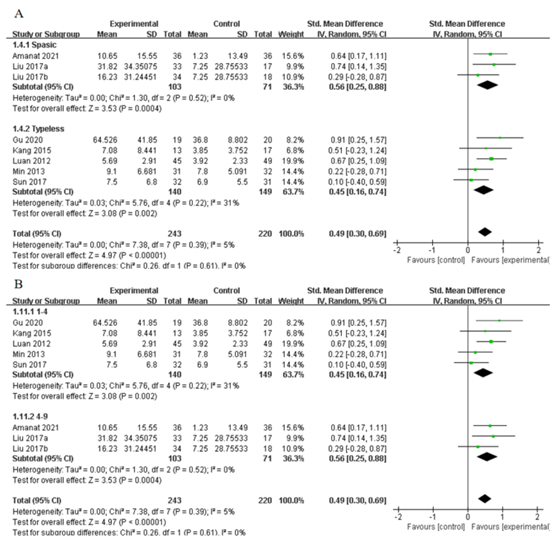

Supplement: Supplementary file 3 [file Image2.TIF]

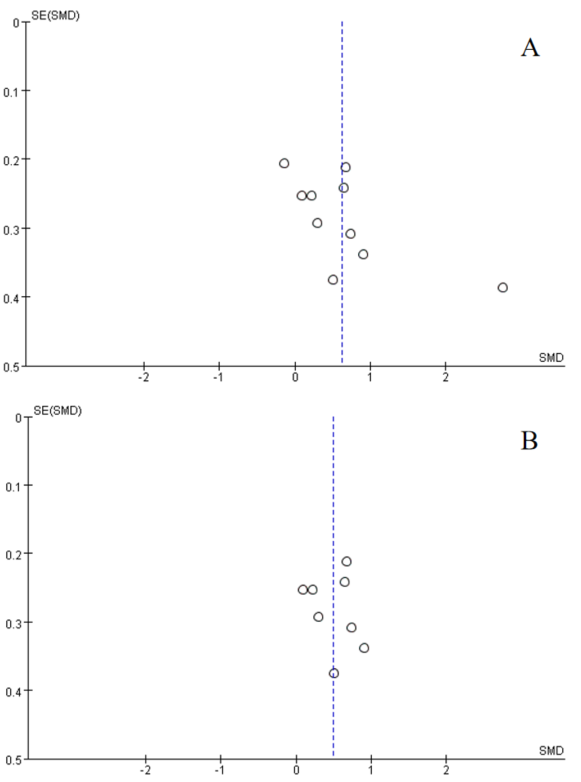

Supplement: Supplementary file 5 [file Image1.TIF]
