# Supplementary material for: Efficacy and safety of stem cell therapy in cerebral palsy: A systematic review and meta-analysis
Source: Front Bioeng Biotechnol. 2022 Dec 14;10:1006845. doi: 10.3389/fbioe.2022.1006845 (PMC9794999; doi:10.3389/fbioe.2022.1006845)
Supplement: Supplementary file 6 [file DataSheet1.PDF]

**Unique ID**	Amanat 2021	**Study ID**	Amanat 2021	**Assessor**	QJY and ZL
**Ref or Label**	https://doi.org/10.1186/s13287-021-02513-4	**Aim**	assignment to intervention (the 'intention-to-treat' effect)		
**Experimental**	Stem cell threapy	**Comparator**	Rehabilitation therapy	**Source**	Journal article(s) with results of the trial; Trial protocol
**Outcome**	GMFM-66	**Results**	mean change 10.65, 95%CI 5.39 to 15.91	**Weight**	1

Domain	Signalling question	Response	Comments
**Bias arising from the randomization process**	1.1 Was the allocation sequence random?	Y	All included participants were randomly assigned in 1:1 ratio using permuted block randomization via interactive web response system to receive either UCT-MSC or sham procedure, respectively.
	1.2 Was the allocation sequence concealed until participants were enrolled and assigned to interventions?	Y	
	1.3 Did baseline differences between intervention groups suggest a problem with the randomization process?	N	Data showed that there were no differences between groups regarding the baseline demographic data (Table 1).
	**Risk of bias judgement**	**Low**	
**Bias due to deviations from intended interventions**	2.1.Were participants aware of their assigned intervention during the trial?	N	The responsible statistician was masked to the clinical data of cases. Personnel staff responsible for cell preparations was not masked, but they had no contacts with participants, parents, or investigators. They also had no information about the clinical and imaging characteristics of patients. All participants, their parents, and investigators were blinded during the study unless serious adverse events occurred that emergent evaluations and treatments by medical staff were essential.
	2.2.Were carers and people delivering the interventions aware of participants' assigned intervention during the trial?	N	
	2.3. If Y/PY/NI to 2.1 or 2.2: Were there deviations from the intended intervention that arose because of the experimental context?	NA	
	2.4 If Y/PY to 2.3: Were these deviations likely to have affected the outcome?	NA	
	2.5. If Y/PY/NI to 2.4: Were these deviations from intended intervention balanced between groups?	NA	
	2.6 Was an appropriate analysis used to estimate the effect of assignment to intervention?	Y	Intention to treat approach was used and all participants who were randomized were included in the statistical analysis.
	2.7 If N/PN/NI to 2.6: Was there potential for a substantial impact (on the result) of the failure to analyse participants in the group to which they were randomized?	NA	
	**Risk of bias judgement**	**Low**	
**Bias due to missing outcome data**	3.1 Were data for this outcome available for all, or nearly all, participants randomized?	PN	Primary screening to identify eligible participants was performed on 321 individuals, and 72 cases were randomly assigned to study arms (36 cases in each group). There were 5 cases (6.9%) who discontinued the study due to the lost to follow-up (n= 3 or 4.1%) or withdrawal of consent (n= 2 or 2.8%).
	3.2 If N/PN/NI to 3.1: Is there evidence that result was not biased by missing outcome data?	PY	One patient was lost to follow-up in experimental group and two in control group.The study was terminated in 1 case in each group.So the number of lost visits was evenly distributed between the two groups.
	3.3 If N/PN to 3.2: Could missingness in the outcome depend on its true value?	NA	
	3.4 If Y/PY/NI to 3.3: Is it likely that missingness in the outcome depended on its true value?	NA	
	**Risk of bias judgement**	**Low**	
**Bias in measurement of the outcome**	4.1 Was the method of measuring the outcome inappropriate?	N	The GMFM-66 was found through Rasch analysis to best describe the gross motor function of children with CP of varying abilities and is a 66 item subset of the original 88 items [29]. It has a unidimensional scale providing interval scaling rather than the ordinal scaling of the GMFM-88. It was shown that inter-rater reliability of Farsi version of this scale for all dimensions was between 0.97 and 0.99 and the intra-rater reliability was 0.99 [30]. Cronbach's alpha coefficient for all dimensions was between 0.78 and 0.94 [30].
	4.2 Could measurement or ascertainment of the outcome have differed between intervention groups?	N	
	4.3 Were outcome assessors aware of the intervention received by study participants?	N	and investigators were blinded during the study unless serious adverse events occurred that emergent evaluations and treatments by medical staff were essential.
	4.4 If Y/PY/NI to 4.3: Could assessment of the outcome have been influenced by knowledge of intervention received?	NA	
	4.5 If Y/PY/NI to 4.4: Is it likely that assessment of the outcome was influenced by knowledge of intervention received?	NA	

	**Risk of bias judgement**	**Low**	
**Bias in selection of the reported result**	5.1 Were the data that produced this result analysed in accordance with a pre-specified analysis plan that was finalized before unblinded outcome data were available for analysis?	Y	ClinicalTrials.gov (NCT03795974)
	5.2 ... multiple eligible outcome measurements (e.g. scales, definitions, time points) within the outcome domain?	N	
	5.3 ... multiple eligible analyses of the data?	N	
	**Risk of bias judgement**	**Low**	
**Overall bias**	**Risk of bias judgement**	**Low**	

**Unique ID**	Gu 2020	**Study ID**	Gu 2020	**Assessor**	QJY and ZL
**Ref or Label**	https://doi.org/10.1186/s13287-019-1545-x	**Aim**	assignment to intervention (the 'intention-to-treat' effect)		
**Experimental**	Stem cell therapy	**Comparator**	Rehabilitation therapy	**Source**	Journal article(s) with results of the trial; Trial protocol
**Outcome**	gross motor function measure (GMFM)	**Results**	64.526 ± 9.600, mean ± SEM	**Weight**	1

Domain	Signalling question	Response	Comments
**Bias arising from the randomization process**	1.1 Was the allocation sequence random?	Y	A randomized block design was utilized with 10 participants in a block. The eligible patients were assigned to one of two groups at a 1:1 ratio according to a random number table, which was generated by a biostatistician with SAS 9.0. To facilitate the blinding procedure, randomization information was sealed in opaque envelopes and delivered to the authorized investigator according to the sequence of screening.
	1.2 Was the allocation sequence concealed until participants were enrolled and assigned to interventions?	Y	
	1.3 Did baseline differences between intervention groups suggest a problem with the randomization process?	N	With respect to the demographics of patients at baseline, no significant differences were found between groups.
	**Risk of bias judgement**	**Low**	
**Bias due to deviations from intended interventions**	2.1.Were participants aware of their assigned intervention during the trial?	N	The patients and their families, as well as the investigators, were all blinded to the grouping information.
	2.2.Were carers and people delivering the interventions aware of participants' assigned intervention during the trial?	N	
	2.3. If Y/PY/NI to 2.1 or 2.2: Were there deviations from the intended intervention that arose because of the experimental context?	NA	
	2.4 If Y/PY to 2.3: Were these deviations likely to have affected the outcome?	NA	
	2.5. If Y/PY/NI to 2.4: Were these deviations from intended intervention balanced between groups?	NA	
	2.6 Was an appropriate analysis used to estimate the effect of assignment to intervention?	Y	Based on our pilot studies, the sample size was calculated based on the assumption that clinical benefit was achieved for 60% of patients in the hUC-MSC group and 10% of patients in the control group (α = 0.05, β = 0.10, allocation ratio 1:1, by two-tailed tests). Thus, at least 14 subjects were required for each group. Therefore, 20 participants were planned for each group considering the withdrawal rate.
	2.7 If N/PN/NI to 2.6: Was there potential for a substantial impact (on the result) of the failure to analyse participants in the group to which they were randomized?	NA	
	**Risk of bias judgement**	**Low**	
**Bias due to missing outcome data**	3.1 Were data for this outcome available for all, or nearly all, participants randomized?	Y	In total, 40 patients were recruited from 8 August 2014 to 31 December 2016, while 1 patient withdrew informed consent without any treatment and was lost to follow-up (Fig. 2). Therefore, 39 patients completed all the study assessments.
	3.2 If N/PN/NI to 3.1: Is there evidence that result was not biased by missing outcome data?	NA	
	3.3 If N/PN to 3.2: Could missingness in the outcome depend on its true value?	NA	
	3.4 If Y/PY/NI to 3.3: Is it likely that missingness in the outcome depended on its true value?	NA	
	**Risk of bias judgement**	**Low**	
**Bias in measurement of the outcome**	4.1 Was the method of measuring the outcome inappropriate?	N	GMFM-88 scale was used to assess gross motor ability regarding “lying and rolling,” “ sitting,” “crawling and kneeling, ” “standing,” and “walking, running, and jumping.”
	4.2 Could measurement or ascertainment of the outcome have differed between intervention groups?	N	
	4.3 Were outcome assessors aware of the intervention received by study participants?	N	The patients and their families, as well as the investigators, were all blinded to the grouping information.

	4.4 If Y/PY/NI to 4.3: Could assessment of the outcome have been influenced by knowledge of intervention received?	NA	
	4.5 If Y/PY/NI to 4.4: Is it likely that assessment of the outcome was influenced by knowledge of intervention received?	NA	
	**Risk of bias judgement**	**Low**	
**Bias in selection of the reported result**	5.1 Were the data that produced this result analysed in accordance with a pre-specified analysis plan that was finalized before unblinded outcome data were available for analysis?	N	Chictr.org.cn, ChiCTR1800016554. Registered 08 June 2018—retrospectively registered. The public title was "Randomized trial of umbilical cord-derived mesenchymal stem cells for cerebral palsy."
	5.2 ... multiple eligible outcome measurements (e.g. scales, definitions, time points) within the outcome domain?	N	
	5.3 ... multiple eligible analyses of the data?	N	
	**Risk of bias judgement**	**Some concerns**	
**Overall bias**	**Risk of bias judgement**	**Some concerns**	

**Unique ID**	Huang 2018	**Study ID**	Huang 2018	**Assessor**	QJY and ZL
**Ref or Label**	Cell Transplantation 2018, Vol. 27(2) 325–334	**Aim**	assignment to intervention (the 'intention-to-treat' effect)		
**Experimental**	Stem cell therapy	**Comparator**	Rehabilitation therapy	**Source**	Journal article(s) with results of the trial
**Outcome**	GMFM-88	**Results**	12.66 ± 0.66, mean ± standard error	**Weight**	1

**Domain**	**Signalling question**	**Response**	**Comments**
**Bias arising from the randomization process**	1.1 Was the allocation sequence random?	Y	All of the patients were randomly assigned to 2 groups on a 1:1 allocation.
	1.2 Was the allocation sequence concealed until participants were enrolled and assigned to interventions?	PY	
	1.3 Did baseline differences between intervention groups suggest a problem with the randomization process?	N	No considerable difference was observed in baseline functional assessments between the 2 groups, including GMFM88 scale scores and the CFA scores.
	**Risk of bias judgement**	**Low**	
**Bias due to deviations from intended interventions**	2.1.Were participants aware of their assigned intervention during the trial?	N	All of the patients and their families were blinded to the group assignment, and the patients received hUCB-MSC infusion with basic rehabilitation in the infusion group, whereas patients in the placebocontrolled group received basic rehabilitation and normal saline (0.9% NS).The investigators and charge nurses were made aware of the treatment information to handle emergen-cies, if any.
	2.2.Were carers and people delivering the interventions aware of participants' assigned intervention during the trial?	Y	
	2.3. If Y/PY/NI to 2.1 or 2.2: Were there deviations from the intended intervention that arose because of the experimental context?	N	Except for 2 patients who dropped out and were lost to follow-up without efficacy assessments, 54 patients in total completed all the required study evaluations at scheduled time points and were included in the statistical analyses.
	2.4 If Y/PY to 2.3: Were these deviations likely to have affected the outcome?	NA	
	2.5. If Y/PY/NI to 2.4: Were these deviations from intended intervention balanced between groups?	NA	
	2.6 Was an appropriate analysis used to estimate the effect of assignment to intervention?	PN	
	2.7 If N/PN/NI to 2.6: Was there potential for a substantial impact (on the result) of the failure to analyse participants in the group to which they were randomized?	PN	
	**Risk of bias judgement**	**Some concerns**	
**Bias due to missing outcome data**	3.1 Were data for this outcome available for all, or nearly all, participants randomized?	Y	Except for 2 patients who dropped out and were lost to follow-up without efficacy assessments, 54 patients in total completed all the required study evaluations at scheduled time points and were included in the statistical analyses.
	3.2 If N/PN/NI to 3.1: Is there evidence that result was not biased by missing outcome data?	NA	
	3.3 If N/PN to 3.2: Could missingness in the outcome depend on its true value?	NA	
	3.4 If Y/PY/NI to 3.3: Is it likely that missingness in the outcome depended on its true value?	NA	
	**Risk of bias judgement**	**Low**	
**Bias in measurement of the outcome**	4.1 Was the method of measuring the outcome inappropriate?	N	
	4.2 Could measurement or ascertainment of the outcome have differed between intervention groups?	N	
	4.3 Were outcome assessors aware of the intervention received by study participants?	PY	
	4.4 If Y/PY/NI to 4.3: Could assessment of the outcome have been influenced by knowledge of intervention received?	PY	
	4.5 If Y/PY/NI to 4.4: Is it likely that assessment of the outcome was influenced by knowledge of intervention received?	PY	

	**Risk of bias judgement**	**High**	
**Bias in selection of the reported result**	5.1 Were the data that produced this result analysed in accordance with a pre-specified analysis plan that was finalized before unblinded outcome data were available for analysis?	NI	No information about clinical registration was found in the article.
	5.2 ... multiple eligible outcome measurements (e.g. scales, definitions, time points) within the outcome domain?	N	
	5.3 ... multiple eligible analyses of the data?	N	
	**Risk of bias judgement**	**Some concerns**	
**Overall bias**	**Risk of bias judgement**	**High**	

**Unique ID**	Kang 2015	**Study ID**	Kang 2015	**Assessor**	QJY and ZL
**Ref or Label**	doi: 10.1089/scd.2015.0074	**Aim**	assignment to intervention (the 'intention-to-treat' effect)		
**Experimental**	Stem cell therapy	**Comparator**	placebo-controlled Active, Rehabilitation	**Source**	Journal article(s) with results of the trial; Trial protocol
**Outcome**	GMFM-88	**Results**	7.08 (2.04)	**Weight**	1

**Domain**	**Signalling question**	**Response**	**Comments**
**Bias arising from the randomization process**	1.1 Was the allocation sequence random?	Y	This study was designed as a placebo-controlled, double-blind study and conducted in accordance with the Declaration of Helsinki. An independent statistician produced a randomization table for patient grouping using SAS software. Subjects were randomly assigned (1:1) into the UCB or control group in accordance with this randomization table.
	1.2 Was the allocation sequence concealed until participants were enrolled and assigned to interventions?	Y	
	1.3 Did baseline differences between intervention groups suggest a problem with the randomization process?	N	Thirty-six children with CP participated in the study, and 34 subjects completed all of the testing procedures (Supplementary Fig. 2). The general baseline characteristics of the UCB and control groups were not significantly different (Table 1). Also, no serious adverse events occurred during this study (Supplementary Table 1).
	**Risk of bias judgement**	**Low**	
**Bias due to deviations from intended interventions**	2.1.Were participants aware of their assigned intervention during the trial?	N	Investigators, assessors, participants, and their parents were all blinded to the group allocation until the study was completed.
	2.2.Were carers and people delivering the interventions aware of participants' assigned intervention during the trial?	N	
	2.3. If Y/PY/NI to 2.1 or 2.2: Were there deviations from the intended intervention that arose because of the experimental context?	NA	
	2.4 If Y/PY to 2.3: Were these deviations likely to have affected the outcome?	NA	
	2.5. If Y/PY/NI to 2.4: Were these deviations from intended intervention balanced between groups?	NA	
	2.6 Was an appropriate analysis used to estimate the effect of assignment to intervention?	N	Power analysis to determine the sample size was not conducted due to the limited number of participating subjects.
	2.7 If N/PN/NI to 2.6: Was there potential for a substantial impact (on the result) of the failure to analyse participants in the group to which they were randomized?	PN	
	**Risk of bias judgement**	**Some concerns**	
**Bias due to missing outcome data**	3.1 Were data for this outcome available for all, or nearly all, participants randomized?	Y	Thirty-six children with CP participated in the study, and 34 subjects completed all of the testing procedures (Supplementary Fig. 2).
	3.2 If N/PN/NI to 3.1: Is there evidence that result was not biased by missing outcome data?	NA	
	3.3 If N/PN to 3.2: Could missingness in the outcome depend on its true value?	NA	
	3.4 If Y/PY/NI to 3.3: Is it likely that missingness in the outcome depended on its true value?	NA	
	**Risk of bias judgement**	**Low**	
**Bias in measurement of the outcome**	4.1 Was the method of measuring the outcome inappropriate?	N	
	4.2 Could measurement or ascertainment of the outcome have differed between intervention groups?	N	
	4.3 Were outcome assessors aware of the intervention received by study participants?	N	Investigators, assessors, participants, and their parents were all blinded to the group allocation until the study was completed.
	4.4 If Y/PY/NI to 4.3: Could assessment of the outcome have been influenced by knowledge of intervention received?	NA	
	4.5 If Y/PY/NI to 4.4: Is it likely that assessment of the outcome was influenced by knowledge of intervention received?	NA	
	**Risk of bias judgement**	**Low**	
**Bias in selection**	5.1 Were the data that produced this result analysed in accordance with a pre-specified analysis plan that was finalized before unblinded outcome data were available for analysis?	Y	This study was approved by the Institutional Review Board and Ethics Committee of CHA Bundang Medical Center and was registered at www.clinicaltrials.gov(NCT01528436).

of the reported result	5.2 ... multiple eligible outcome measurements (e.g. scales, definitions, time points) within the outcome domain?	N	
	5.3 ... multiple eligible analyses of the data?	N	
	**Risk of bias judgement**	**Low**	
**Overall bias**	**Risk of bias judgement**	**Some concerns**	

**Unique ID**	Liu 2017	**Study ID**	Liu 2017	**Assessor**	QJY and ZL
**Ref or Label**	J Transl Med (2017) 15:48	**Aim**	assignment to intervention (the 'intention-to-treat' effect)		
**Experimental**	Stem cell therapy	**Comparator**	Rehabilitation therapy	**Source**	Journal article(s) with results of the trial; Trial protocol
**Outcome**	GMFM	**Results**	127.03 ± 35.80 and 111.91 ± 31.68	**Weight**	1

Domain	Signalling question	Response	Comments
**Bias arising from the randomization process**	1.1 Was the allocation sequence random?	Y	This study involved 105 CP patients who were enrolled from May 1, 2010, to October 31, 2012. Patients were randomly assigned into the BMMSC group, the BMMNC group or the control group in a 1:1:1 ratio. The randomisation table was generated by SAS software. After randomisation, the study processes were blinded to the patients in the BMMSC and BMMNC groups, participant surgeons, coordinators, and the investigators who were responsible for patient assessment.
	1.2 Was the allocation sequence concealed until participants were enrolled and assigned to interventions?	Y	
	1.3 Did baseline differences between intervention groups suggest a problem with the randomization process?	N	In comparison of the baseline of GMFM, FMFM, and dimensions among the BMMCS group, BMMNS group, and the control group, the differences were not statistically significant (P > 0.05)
	**Risk of bias judgement**	**Low**	
**Bias due to deviations from intended interventions**	2.1.Were participants aware of their assigned intervention during the trial?	N	The randomisation table was generated by SAS software. After randomisation, the study processes were blinded to the patients in the BMMSC and BMMNC groups, participant surgeons, coordinators, and the investigators who were responsible for patient assessment.
	2.2.Were carers and people delivering the interventions aware of participants' assigned intervention during the trial?	N	
	2.3. If Y/PY/NI to 2.1 or 2.2: Were there deviations from the intended intervention that arose because of the experimental context?	NA	
	2.4 If Y/PY to 2.3: Were these deviations likely to have affected the outcome?	NA	
	2.5. If Y/PY/NI to 2.4: Were these deviations from intended intervention balanced between groups?	NA	
	2.6 Was an appropriate analysis used to estimate the effect of assignment to intervention?	NI	
	2.7 If N/PN/NI to 2.6: Was there potential for a substantial impact (on the result) of the failure to analyse participants in the group to which they were randomized?	PN	
	**Risk of bias judgement**	**Some concerns**	
**Bias due to missing outcome data**	3.1 Were data for this outcome available for all, or nearly all, participants randomized?	Y	Two children in the BMMSC group and one child in the BMMNC group left the experiment due to their parent withdrawal. In all, there are 33 patients in the BMMSC group (18 boys and 15 girls), and 34 patients (18 boys and 16 girls) in the BMMNC group completed the experiment. The general details, as well as GMFM and FMFM scores are provided in Additional file 1, Tables 1 and 2.
	3.2 If N/PN/NI to 3.1: Is there evidence that result was not biased by missing outcome data?	NA	
	3.3 If N/PN to 3.2: Could missingness in the outcome depend on its true value?	NA	
	3.4 If Y/PY/NI to 3.3: Is it likely that missingness in the outcome depended on its true value?	NA	
	**Risk of bias judgement**	**Low**	
**Bias in measurement of the outcome**	4.1 Was the method of measuring the outcome inappropriate?	N	The gross motor function measure (GMFM) and fine motor function measure (FMFM) were used to evaluate the efficacy of cell therapy.
	4.2 Could measurement or ascertainment of the outcome have differed between intervention groups?	N	
	4.3 Were outcome assessors aware of the intervention received by study participants?	N	After randomisation, the study processes were blinded to the patients in the BMMSC and BMMNC groups, participant surgeons, coordinators, and the investigators who were responsible for patient assessment.

	4.4 If Y/PY/NI to 4.3: Could assessment of the outcome have been influenced by knowledge of intervention received?	NA	
	4.5 If Y/PY/NI to 4.4: Is it likely that assessment of the outcome was influenced by knowledge of intervention received?	NA	
	**Risk of bias judgement**	**Low**	
**Bias in selection of the reported result**	5.1 Were the data that produced this result analysed in accordance with a pre-specified analysis plan that was finalized before unblinded outcome data were available for analysis?	Y	The study was approved by the General Hospital of Chinese People's Armed Police Forces Medical Ethics Committee and has been registered in WHO (registration number CHiCTR-TRC-12002568).
	5.2 ... multiple eligible outcome measurements (e.g. scales, definitions, time points) within the outcome domain?	N	
	5.3 ... multiple eligible analyses of the data?	N	
	**Risk of bias judgement**	**Low**	
**Overall bias**	**Risk of bias judgement**	**Some concerns**	

**Unique ID**	Luan 2012	**Study ID**	Luan 2012	**Assessor**	QJY and ZL
**Ref or Label**	Cell Transplantation, Vol. 21, Supplement 1, pp. S91–S98, 2012	**Aim**	assignment to intervention (the 'intention-to-treat' effect)		
**Experimental**	Stem cell therapy	**Comparator**	Rehabilitation therapy	**Source**	Journal article(s) with results of the trial; Trial protocol
**Outcome**	GMFM	**Results**	12.94 ± 10.93	**Weight**	1

**Domain**	**Signalling question**	**Response**	**Comments**
**Bias arising from the randomization process**	1.1 Was the allocation sequence random?	Y	The included patients were randomly assigned to two groups (treatment group, n = 45; control group, n = 49).
	1.2 Was the allocation sequence concealed until participants were enrolled and assigned to interventions?	PY	
	1.3 Did baseline differences between intervention groups suggest a problem with the randomization process?	N	
	**Risk of bias judgement**	**Low**	
**Bias due to deviations from intended interventions**	2.1.Were participants aware of their assigned intervention during the trial?	PY	We don't see any explanation in the article about the practice of blinding.
	2.2.Were carers and people delivering the interventions aware of participants' assigned intervention during the trial?	PY	
	2.3. If Y/PY/NI to 2.1 or 2.2: Were there deviations from the intended intervention that arose because of the experimental context?	N	
	2.4 If Y/PY to 2.3: Were these deviations likely to have affected the outcome?	NA	
	2.5. If Y/PY/NI to 2.4: Were these deviations from intended intervention balanced between groups?	NA	
	2.6 Was an appropriate analysis used to estimate the effect of assignment to intervention?	NI	
	2.7 If N/PN/NI to 2.6: Was there potential for a substantial impact (on the result) of the failure to analyse participants in the group to which they were randomized?	PN	
	**Risk of bias judgement**	**Some concerns**	
**Bias due to missing outcome data**	3.1 Were data for this outcome available for all, or nearly all, participants randomized?	Y	A total of 94 consecutive patients visiting our hospital who met the following criteria were recruited.
	3.2 If N/PN/NI to 3.1: Is there evidence that result was not biased by missing outcome data?	NA	
	3.3 If N/PN to 3.2: Could missingness in the outcome depend on its true value?	NA	
	3.4 If Y/PY/NI to 3.3: Is it likely that missingness in the outcome depended on its true value?	NA	
	**Risk of bias judgement**	**Low**	
**Bias in measurement of the outcome**	4.1 Was the method of measuring the outcome inappropriate?	N	
	4.2 Could measurement or ascertainment of the outcome have differed between intervention groups?	N	
	4.3 Were outcome assessors aware of the intervention received by study participants?	PY	
	4.4 If Y/PY/NI to 4.3: Could assessment of the outcome have been influenced by knowledge of intervention received?	PY	
	4.5 If Y/PY/NI to 4.4: Is it likely that assessment of the outcome was influenced by knowledge of intervention received?	PY	
	**Risk of bias judgement**	**High**	
**Bias in selection of the reported result**	5.1 Were the data that produced this result analysed in accordance with a pre-specified analysis plan that was finalized before unblinded outcome data were available for analysis?	PN	The protocol of clinical studies was approved by the Scientific Council and Ethics Committee of Navy Gen-eral Hospital and in accordance with guidelines issued by the Chinese Ministry of Health (91-006)
	5.2 ... multiple eligible outcome measurements (e.g. scales, definitions, time points) within the outcome domain?	PN	
	5.3 ... multiple eligible analyses of the data?	PN	
	**Risk of bias judgement**	**Some concerns**	
**Overall bias**	**Risk of bias judgement**	**High**	

**Unique ID**	Min 2013	**Study ID**	Min 2013	**Assessor**	QJY and ZL
**Ref or Label**	STEM CELLS 2013;31:581–591	**Aim**	assignment to intervention (the 'intention-to-treat' effect)		
**Experimental**	Stem cell therapy	**Comparator**	Rehabilitation therapy	**Source**	Journal article(s) with results of the trial; Trial protocol
**Outcome**	GMFM	**Results**	14.5 (1.8), mean (SE)	**Weight**	1

**Domain**	**Signalling question**	**Response**	**Comments**
**Bias arising from the randomization process**	1.1 Was the allocation sequence random?	Y	The children were randomly distributed (i.e., 1:1:1 allocation) by an independent provider for each unit who was not informed about each subject.
	1.2 Was the allocation sequence concealed until participants were enrolled and assigned to interventions?	Y	
	1.3 Did baseline differences between intervention groups suggest a problem with the randomization process?	N	There were no significant differences between the three groups, pUCB (n = 31), EPO (n = 33), and Control (n = 32), in the demographic data, MRI findings [41], severity of disease [42], typology [1], residence area, and duration of previous and postdischarge rehabilitation (Table 1; Supporting Information Table S1).
	**Risk of bias judgement**	**Low**	
**Bias due to deviations from intended interventions**	2.1.Were participants aware of their assigned intervention during the trial?	N	In accordance with a placebo-controlled double-blind trial protocol, all participants including family members, observers, investigators, and employees were blinded to the group assignment.
	2.2.Were carers and people delivering the interventions aware of participants' assigned intervention during the trial?	N	
	2.3. If Y/PY/NI to 2.1 or 2.2: Were there deviations from the intended intervention that arose because of the experimental context?	NA	
	2.4 If Y/PY to 2.3: Were these deviations likely to have affected the outcome?	NA	
	2.5. If Y/PY/NI to 2.4: Were these deviations from intended intervention balanced between groups?	NA	
	2.6 Was an appropriate analysis used to estimate the effect of assignment to intervention?	NI	
	2.7 If N/PN/NI to 2.6: Was there potential for a substantial impact (on the result) of the failure to analyse participants in the group to which they were randomized?	PN	
	**Risk of bias judgement**	**Some concerns**	
**Bias due to missing outcome data**	3.1 Were data for this outcome available for all, or nearly all, participants randomized?	PN	Among the 105 children enrolled in this study, nine dropped out (Supporting Information Contents 1). Thus, 96 participants were included in the analyses [illegible]
	3.2 If N/PN/NI to 3.1: Is there evidence that result was not biased by missing outcome data?	PN	
	3.3 If N/PN to 3.2: Could missingness in the outcome depend on its true value?	PN	Among the 105 children enrolled in this study, nine dropped out (Supporting Information Contents 1).
	3.4 If Y/PY/NI to 3.3: Is it likely that missingness in the outcome depended on its true value?	NA	
	**Risk of bias judgement**	**Low**	
**Bias in measurement of the outcome**	4.1 Was the method of measuring the outcome inappropriate?	N	The inter-rater reliability ICCs of the GMFM subscores and total score were 0.97–1.00 (n = 101, 10 raters) and the intrarater reliability ICCs were 0.99–1.00 (n = 101, two raters).
	4.2 Could measurement or ascertainment of the outcome have differed between intervention groups?	N	
	4.3 Were outcome assessors aware of the intervention received by study participants?	N	In accordance with a placebo-controlled double-blind trial protocol, all participants including family members, observers, investigators, and employees were blinded to the group assignment.
	4.4 If Y/PY/NI to 4.3: Could assessment of the outcome have been influenced by knowledge of intervention received?	NA	
	4.5 If Y/PY/NI to 4.4: Is it likely that assessment of the outcome was influenced by knowledge of intervention received?	NA	
	**Risk of bias judgement**	**Low**	
**Bias in selection of the reported result**	5.1 Were the data that produced this result analysed in accordance with a pre-specified analysis plan that was finalized before unblinded outcome data were available for analysis?	Y	This trial was registered at www.clinicaltrials.gov (NCT01193660).
	5.2 ... multiple eligible outcome measurements (e.g. scales, definitions, time points) within the outcome domain?	N	
	5.3 ... multiple eligible analyses of the data?	N	
	**Risk of bias judgement**	**Low**	
**Overall bias**	**Risk of bias judgement**	**Some concerns**	

**Unique ID**	Rah 2017	**Study ID**	Rah 2017	**Assessor**	QJY and ZL

**Ref or Label**	Rah et al. J Transl Med (2017) 15:16	**Aim**	assignment to intervention (the 'intention-to-treat' effect)		
**Experimental**	Stem cell therapy	**Comparator**	Rehabilitation therapy	**Source**	Journal article(s) with results of the trial; Trial protocol
**Outcome**	GMFM	**Results**	0.3725	**Weight**	1

Domain	Signalling question	Response	Comments
**Bias arising from the randomization process**	1.1 Was the allocation sequence random?	Y	One month after cryopreservation of the mPBMCs (M1), patients were randomized to receive either mPBMCs or placebo. We performed a randomized, double-blind, cross-over study to assess the neuroregenerative potential of intravenous granulocyte colony-stimulating factor (G-CSF) followed by infusion of mobilized peripheral blood mono-nuclear cells (mPBMCs) in children with cerebral palsy (CP).
	1.2 Was the allocation sequence concealed until participants were enrolled and assigned to interventions?	Y	
	1.3 Did baseline differences between intervention groups suggest a problem with the randomization process?	NI	
	**Risk of bias judgement**	**Low**	
**Bias due to deviations from intended interventions**	2.1.Were participants aware of their assigned intervention during the trial?	N	One month after cryopreservation of the mPBMCs (M1), patients were randomized to receive either mPBMCs or placebo. Masking:Quadruple (Participant, Care Provider, Investigator, Outcomes Assessor)
	2.2.Were carers and people delivering the interventions aware of participants' assigned intervention during the trial?	N	
	2.3. If Y/PY/NI to 2.1 or 2.2: Were there deviations from the intended intervention that arose because of the experimental context?	NA	
	2.4 If Y/PY to 2.3: Were these deviations likely to have affected the outcome?	NA	
	2.5. If Y/PY/NI to 2.4: Were these deviations from intended intervention balanced between groups?	NA	
	2.6 Was an appropriate analysis used to estimate the effect of assignment to intervention?	NI	
	2.7 If N/PN/NI to 2.6: Was there potential for a substantial impact (on the result) of the failure to analyse participants in the group to which they were randomized?	PN	
	**Risk of bias judgement**	**Some concerns**	
**Bias due to missing outcome data**	3.1 Were data for this outcome available for all, or nearly all, participants randomized?	N	Fifty-seven patients were enrolled in the current study. Forty-seven patients for whom complete study data were available were included in this analysis.
	3.2 If N/PN/NI to 3.1: Is there evidence that result was not biased by missing outcome data?	PN	
	3.3 If N/PN to 3.2: Could missingness in the outcome depend on its true value?	NI	
	3.4 If Y/PY/NI to 3.3: Is it likely that missingness in the outcome depended on its true value?	NI	
	**Risk of bias judgement**	**High**	
**Bias in measurement of the outcome**	4.1 Was the method of measuring the outcome inappropriate?	N	
	4.2 Could measurement or ascertainment of the outcome have differed between intervention groups?	N	The results for each examination tool were evaluated by well-trained physical and occupational therapists, and therapeutic responses were comprehensively assessed by rehabilitation specialists.
	4.3 Were outcome assessors aware of the intervention received by study participants?	N	Masking:Quadruple (Participant, Care Provider, Investigator, Outcomes Assessor)
	4.4 If Y/PY/NI to 4.3: Could assessment of the outcome have been influenced by knowledge of intervention received?	NA	
	4.5 If Y/PY/NI to 4.4: Is it likely that assessment of the outcome was influenced by knowledge of intervention received?	NA	
	**Risk of bias judgement**	**Low**	
**Bias in selection of the reported result**	5.1 Were the data that produced this result analysed in accordance with a pre-specified analysis plan that was finalized before unblinded outcome data were available for analysis?	N	Trial registration: ClinicalTrials.gov, NCT02983708. Registered 5 December, 2016, retrospectively registered
	5.2 ... multiple eligible outcome measurements (e.g. scales, definitions, time points) within the outcome domain?	N	
	5.3 ... multiple eligible analyses of the data?	N	
	**Risk of bias judgement**	**Some concerns**	
**Overall bias**	**Risk of bias judgement**	**High**	

**Unique ID**	Sun 2017	**Study ID**	Sun 2017	**Assessor**	QJY and ZL
**Ref or Label**	STEM CELLS TRANSLATIONAL MEDICINE 2017;6:2071–2078	**Aim**	assignment to intervention (the 'intention-to-treat' effect)		
**Experimental**	Stem cell therapy	**Comparator**	Rehabilitation threapy	**Source**	Journal article(s) with results of the trial; Trial protocol

Outcome	GMFM-66	Results	7.5 points (SD 6.8)	Weight	1

Domain	Signalling question	Response	Comments
**Bias arising from the randomization process**	1.1 Was the allocation sequence random?	Y	Computergenerated randomization was performed by The Emmes Corporation in a 1:1 ratio, stratified by age and CP typography.
	1.2 Was the allocation sequence concealed until participants were enrolled and assigned to interventions?	Y	
	1.3 Did baseline differences between intervention groups suggest a problem with the randomization process?	N	Subjects' etiology of CP was classified as: periventricular leukomalacia (n 5 17), in utero stroke/bleed (n 5 27), ischemic injury (n 5 7), other multifactorial causes doses were not associated with baseline age or type/severity of CP (Table 2).
	**Risk of bias judgement**	**Low**	
**Bias due to deviations from intended interventions**	2.1.Were participants aware of their assigned intervention during the trial?	N	Only staff preparing the products were aware of the treatment assignment, and these individuals had no contact with the patients, families, providers, and examiners who were masked to the assigned treatment. Masking was achieved by covering all infusion bags with a dark bag in the laboratory and infusing a similar volume as the placebo product.
	2.2.Were carers and people delivering the interventions aware of participants' assigned intervention during the trial?	N	
	2.3. If Y/PY/NI to 2.1 or 2.2: Were there deviations from the intended intervention that arose because of the experimental context?	NA	
	2.4 If Y/PY to 2.3: Were these deviations likely to have affected the outcome?	NA	
	2.5. If Y/PY/NI to 2.4: Were these deviations from intended intervention balanced between groups?	NA	
	2.6 Was an appropriate analysis used to estimate the effect of assignment to intervention?	Y	Sample size planning used estimates of this change score in untreated patients derived from a literature review (mean 5 6, SD 5 3) [14, 15].
	2.7 If N/PN/NI to 2.6: Was there potential for a substantial impact (on the result) of the failure to analyse participants in the group to which they were randomized?	NA	
	**Risk of bias judgement**	**Low**	
**Bias due to missing outcome data**	3.1 Were data for this outcome available for all, or nearly all, participants randomized?	Y	
	3.2 If N/PN/NI to 3.1: Is there evidence that result was not biased by missing outcome data?	NA	
	3.3 If N/PN to 3.2: Could missingness in the outcome depend on its true value?	NA	
	3.4 If Y/PY/NI to 3.3: Is it likely that missingness in the outcome depended on its true value?	NA	
	**Risk of bias judgement**	**Low**	
**Bias in measurement of the outcome**	4.1 Was the method of measuring the outcome inappropriate?	N	GMFM-66 [7], a 66-item measure designed to assess gross motor function in children with CP
	4.2 Could measurement or ascertainment of the outcome have differed between intervention groups?	N	
	4.3 Were outcome assessors aware of the intervention received by study participants?	N	Only staff preparing the products were aware of the treatment assignment, and these individuals had no contact with the patients, families, providers, and examiners who were masked to the assigned treatment.
	4.4 If Y/PY/NI to 4.3: Could assessment of the outcome have been influenced by knowledge of intervention received?	NA	
	4.5 If Y/PY/NI to 4.4: Is it likely that assessment of the outcome was influenced by knowledge of intervention received?	NA	
	**Risk of bias judgement**	**Low**	
**Bias in selection of the reported result**	5.1 Were the data that produced this result analysed in accordance with a pre-specified analysis plan that was finalized before unblinded outcome data were available for analysis?	Y	This trial is registered with ClinicalTrials.gov, number NCT01147653.
	5.2 ... multiple eligible outcome measurements (e.g. scales, definitions, time points) within the outcome domain?	N	
	5.3 ... multiple eligible analyses of the data?	N	
	**Risk of bias judgement**	**Low**	
**Overall bias**	**Risk of bias judgement**	**Low**	
